# Supplementary figures and images for: Camel Milk-Derived Extracellular Vesicles as a Functional Food Component Ameliorate Hypobaric Hypoxia-Induced Colonic Injury Through Microbiota–Metabolite Crosstalk
Source: Nutrients. 2025 Jul 25;17(15):2431. doi: 10.3390/nu17152431 (PMC12348715; doi:10.3390/nu17152431)

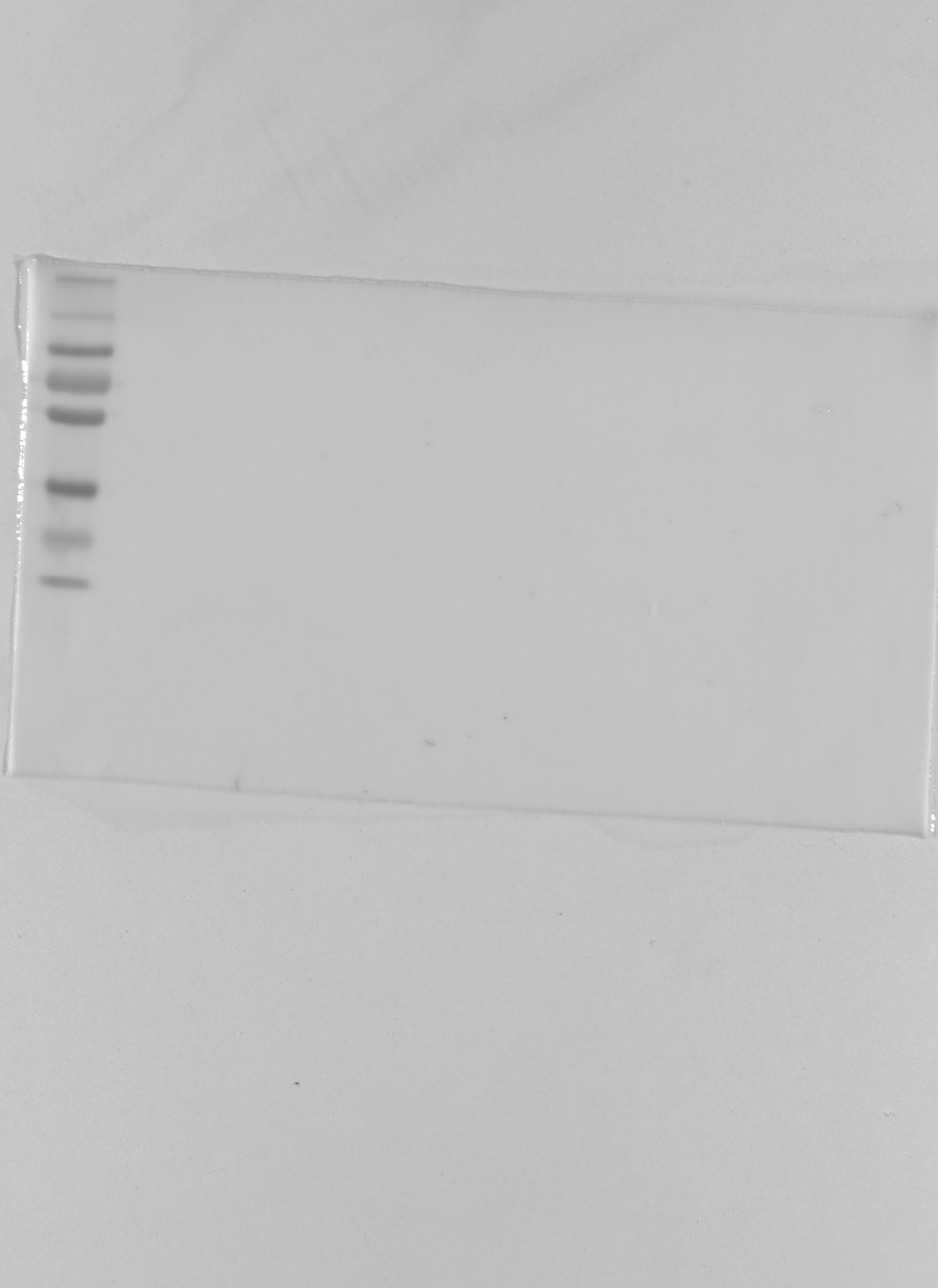

Supplement: Supplementary file 1 [file nutrients-17-02431-s001.zip › colon-WB/6.4 actin 20240604_231923_Ch/6.4 actin 20240604_231923_Ch-Marker.tif]

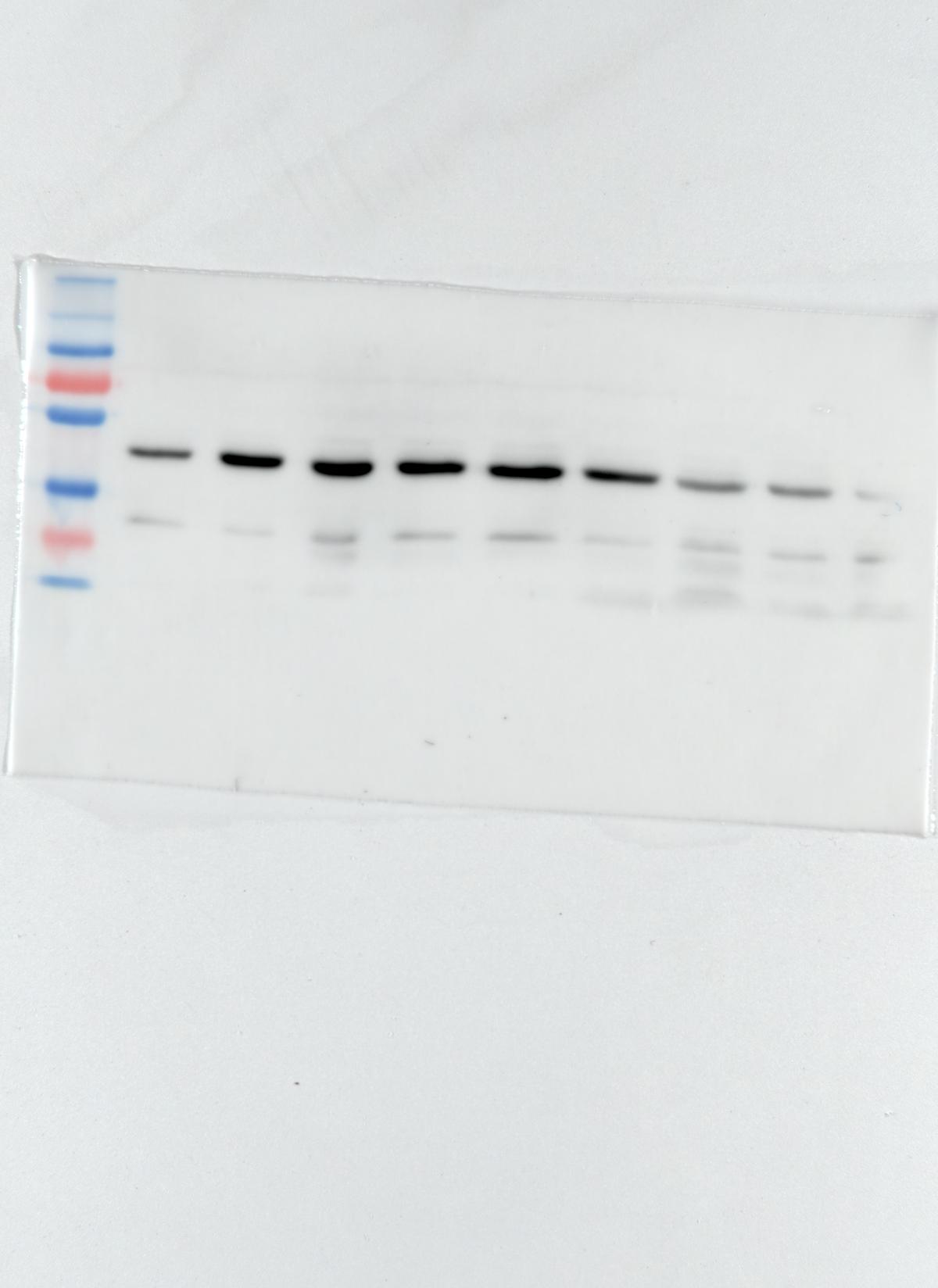

Supplement: Supplementary file 1 [file nutrients-17-02431-s001.zip › colon-WB/6.4 actin 20240604_231923_Ch/6.4 actin 20240604_231923_Ch_Chemi+Marker.jpg]

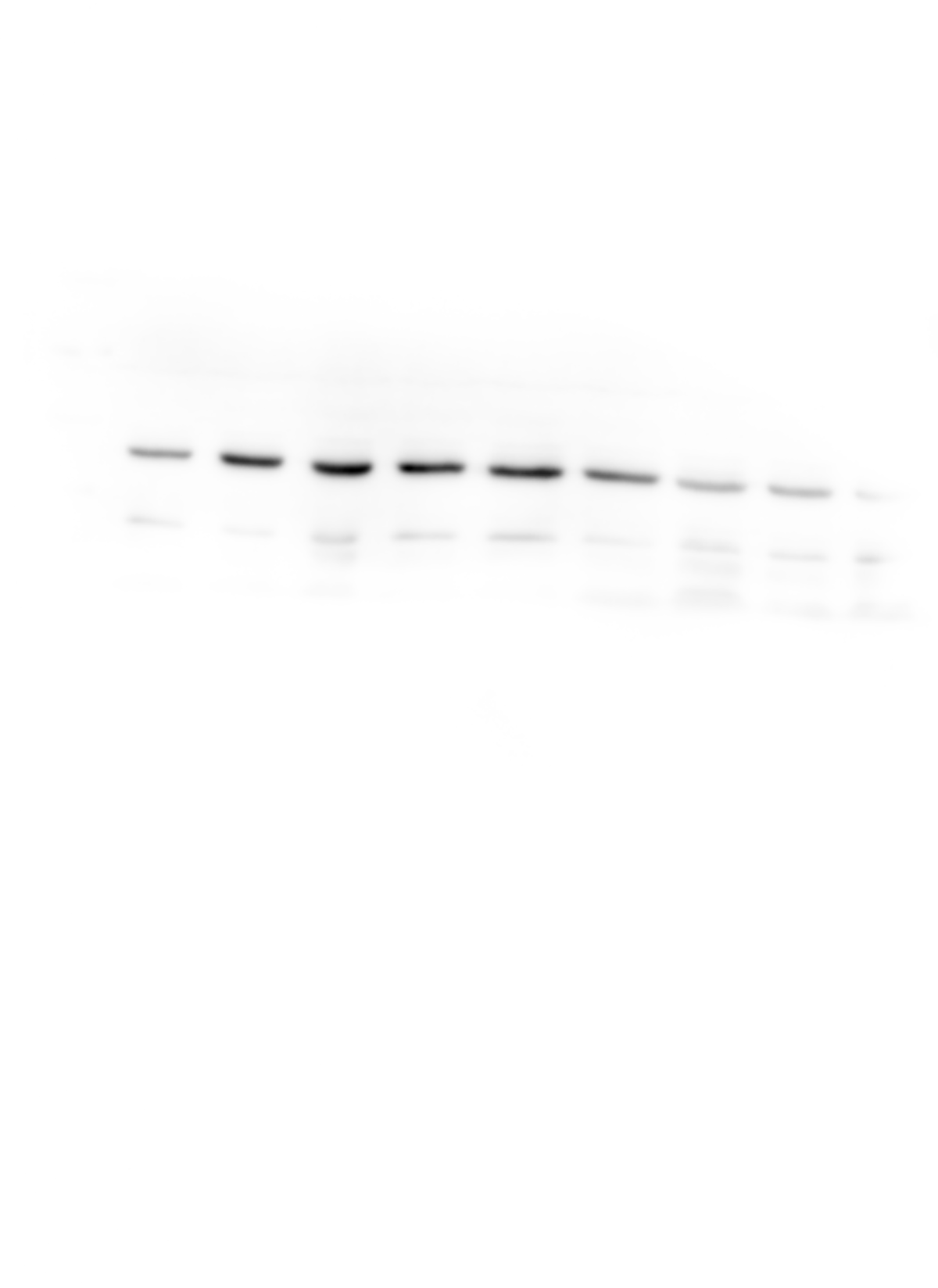

Supplement: Supplementary file 1 [file nutrients-17-02431-s001.zip › colon-WB/6.4 actin 20240604_231923_Ch/6.4 actin 20240604_231923_Ch_Chemi.tif]

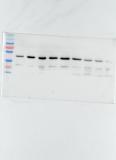

Supplement: Supplementary file 1 [file nutrients-17-02431-s001.zip › colon-WB/6.4 actin 20240604_231923_Ch/6.4 actin 20240604_231923_Ch_Thumb.jpg]

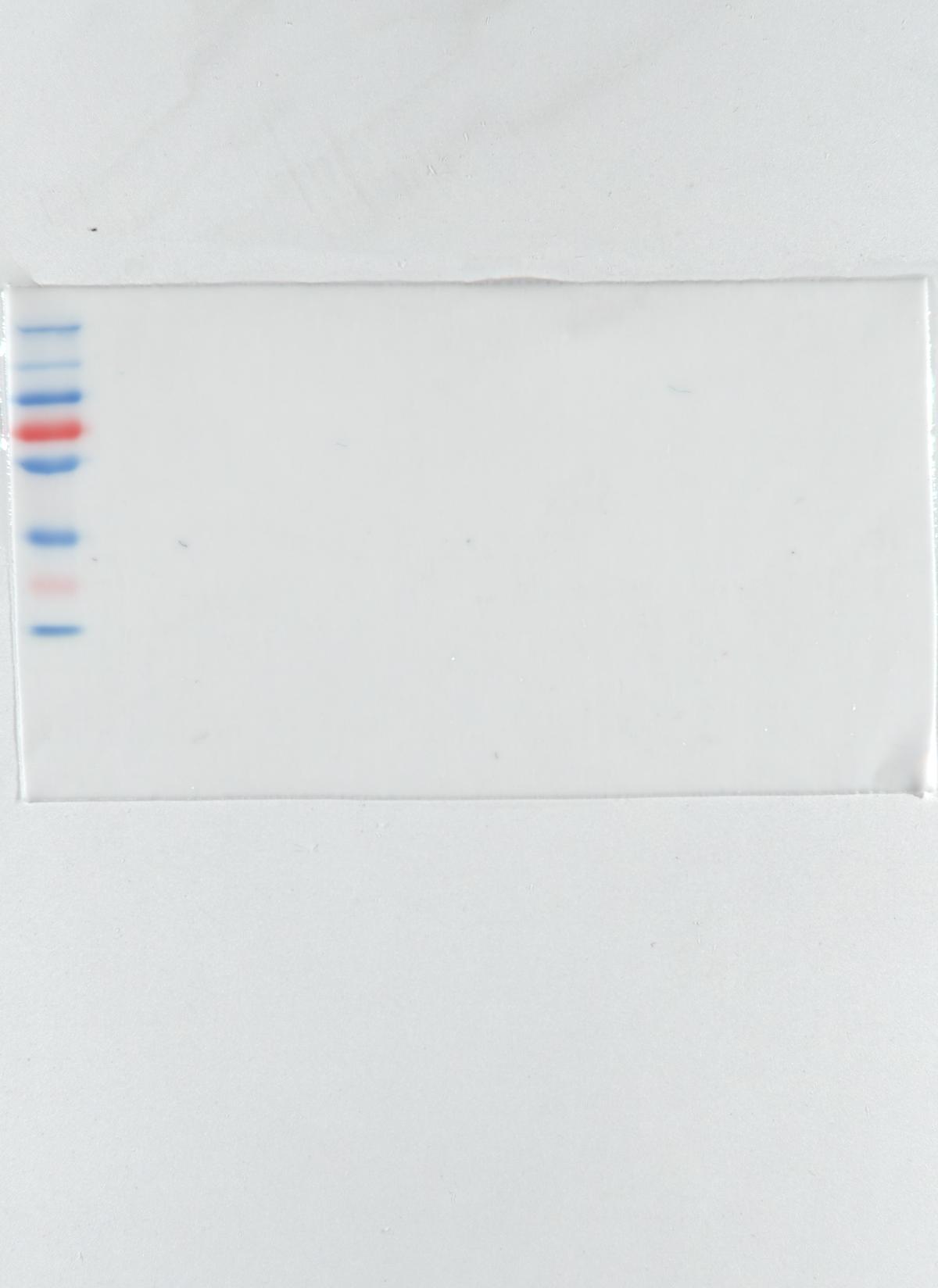

Supplement: Supplementary file 1 [file nutrients-17-02431-s001.zip › colon-WB/6.4 zo1 20240604_232239_Ch/6.4 zo1 20240604_232239_Ch-Marker.jpg]

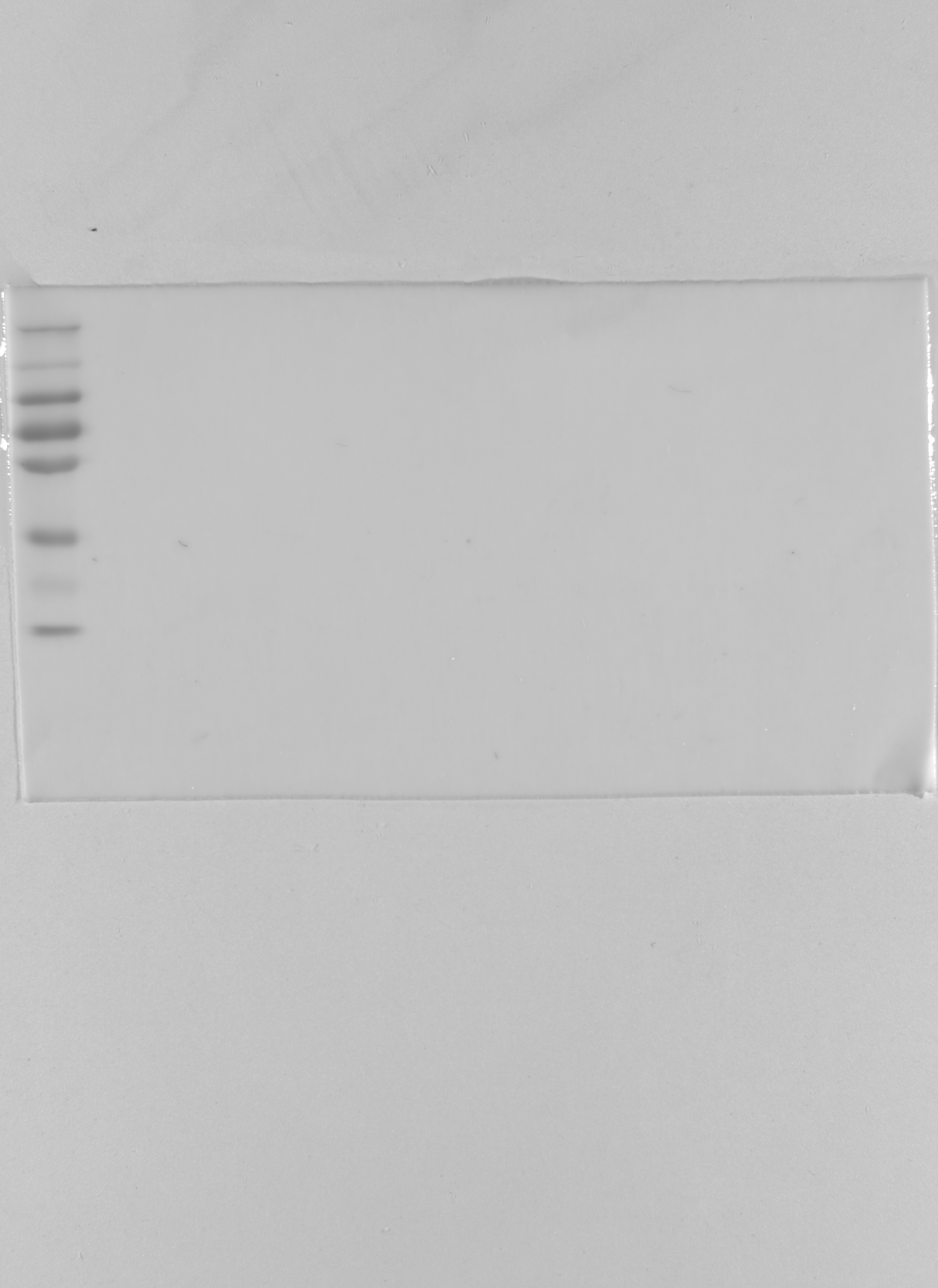

Supplement: Supplementary file 1 [file nutrients-17-02431-s001.zip › colon-WB/6.4 zo1 20240604_232239_Ch/6.4 zo1 20240604_232239_Ch-Marker.tif]

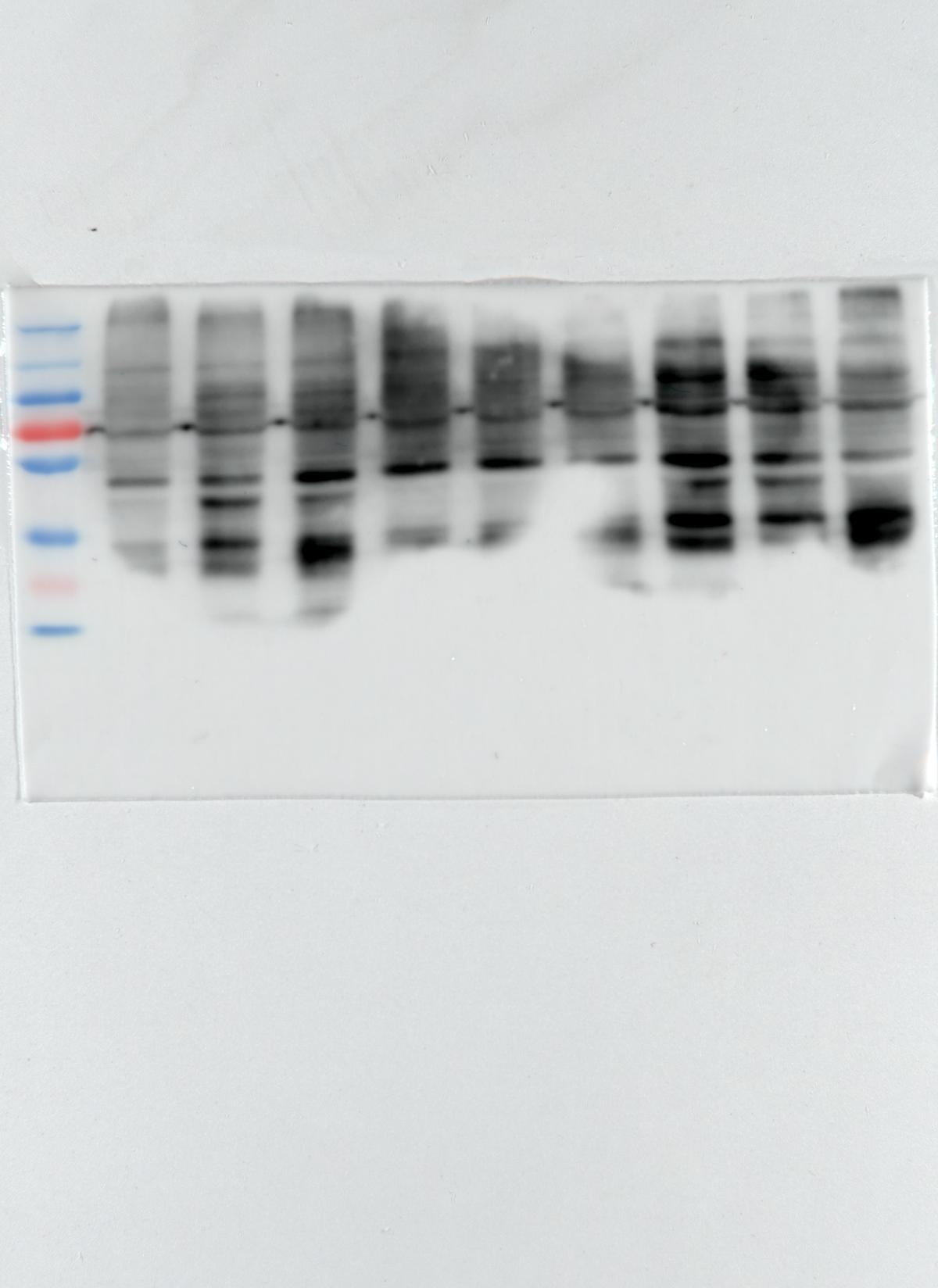

Supplement: Supplementary file 1 [file nutrients-17-02431-s001.zip › colon-WB/6.4 zo1 20240604_232239_Ch/6.4 zo1 20240604_232239_Ch_Chemi+Marker.jpg]

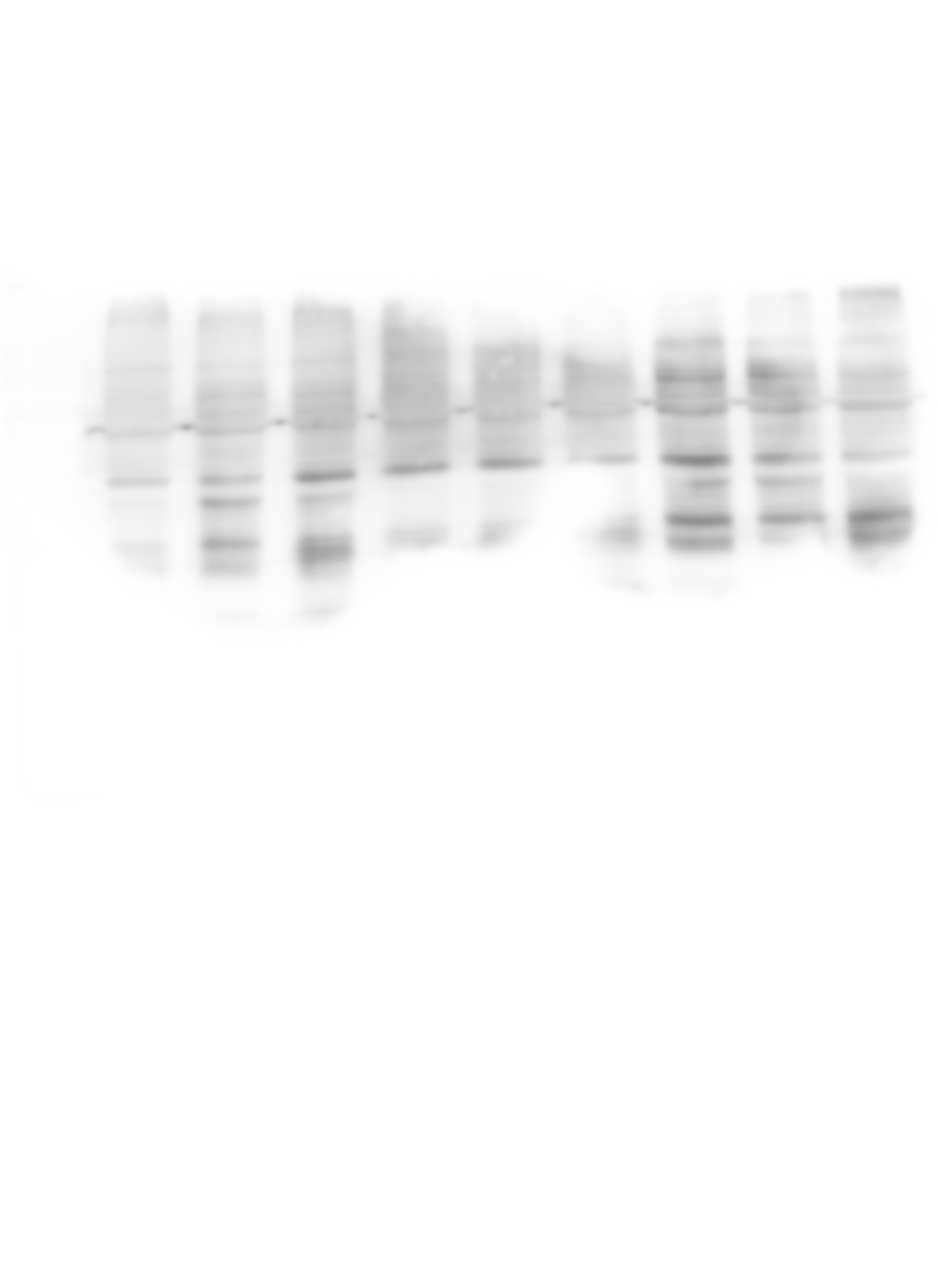

Supplement: Supplementary file 1 [file nutrients-17-02431-s001.zip › colon-WB/6.4 zo1 20240604_232239_Ch/6.4 zo1 20240604_232239_Ch_Chemi.tif]

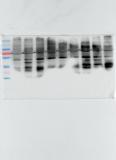

Supplement: Supplementary file 1 [file nutrients-17-02431-s001.zip › colon-WB/6.4 zo1 20240604_232239_Ch/6.4 zo1 20240604_232239_Ch_Thumb.jpg]

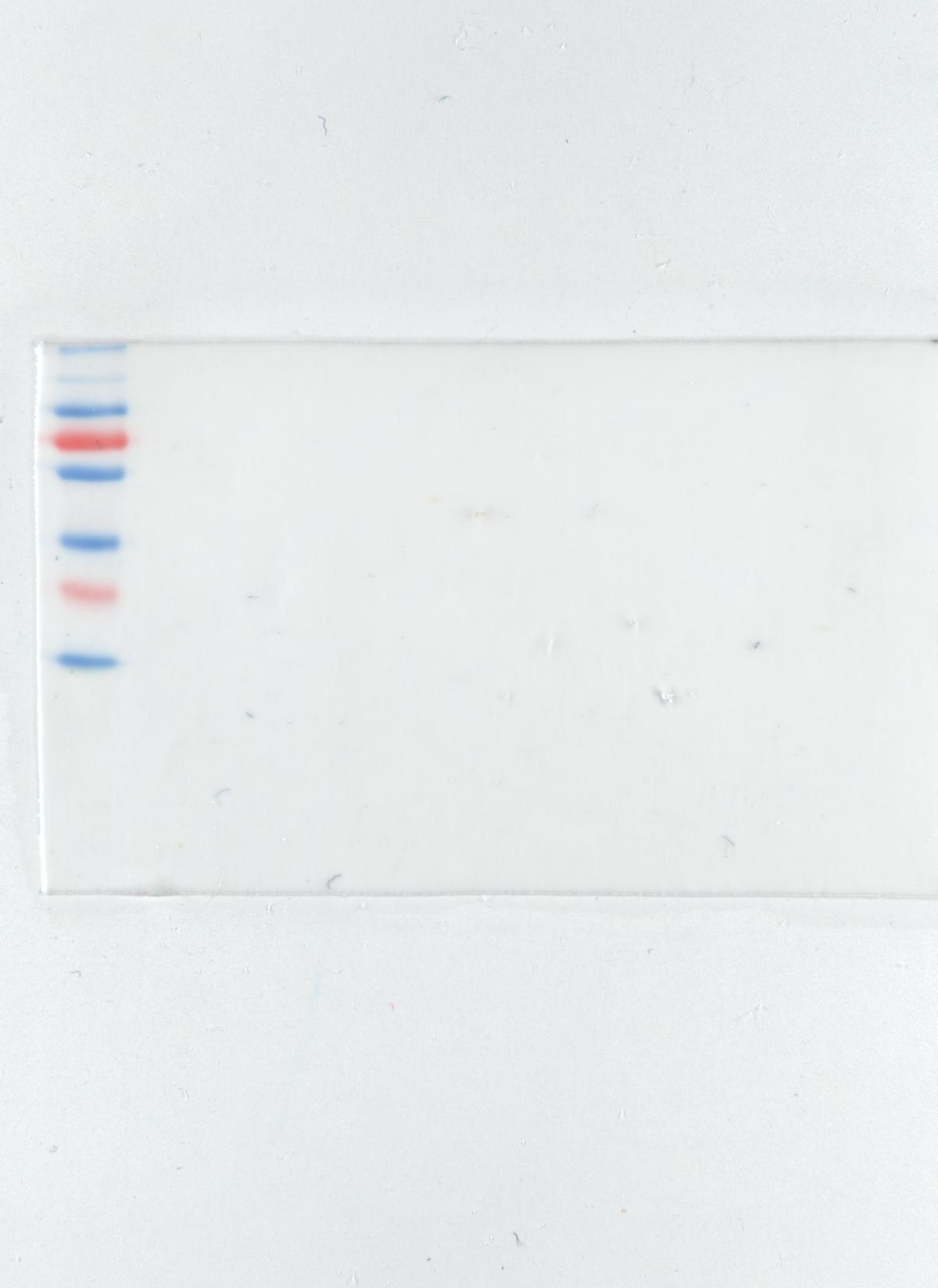

Supplement: Supplementary file 1 [file nutrients-17-02431-s001.zip › colon-WB/6.8 actin 20240608_162313_Ch/6.8 actin 20240608_162313_Ch-Marker.jpg]

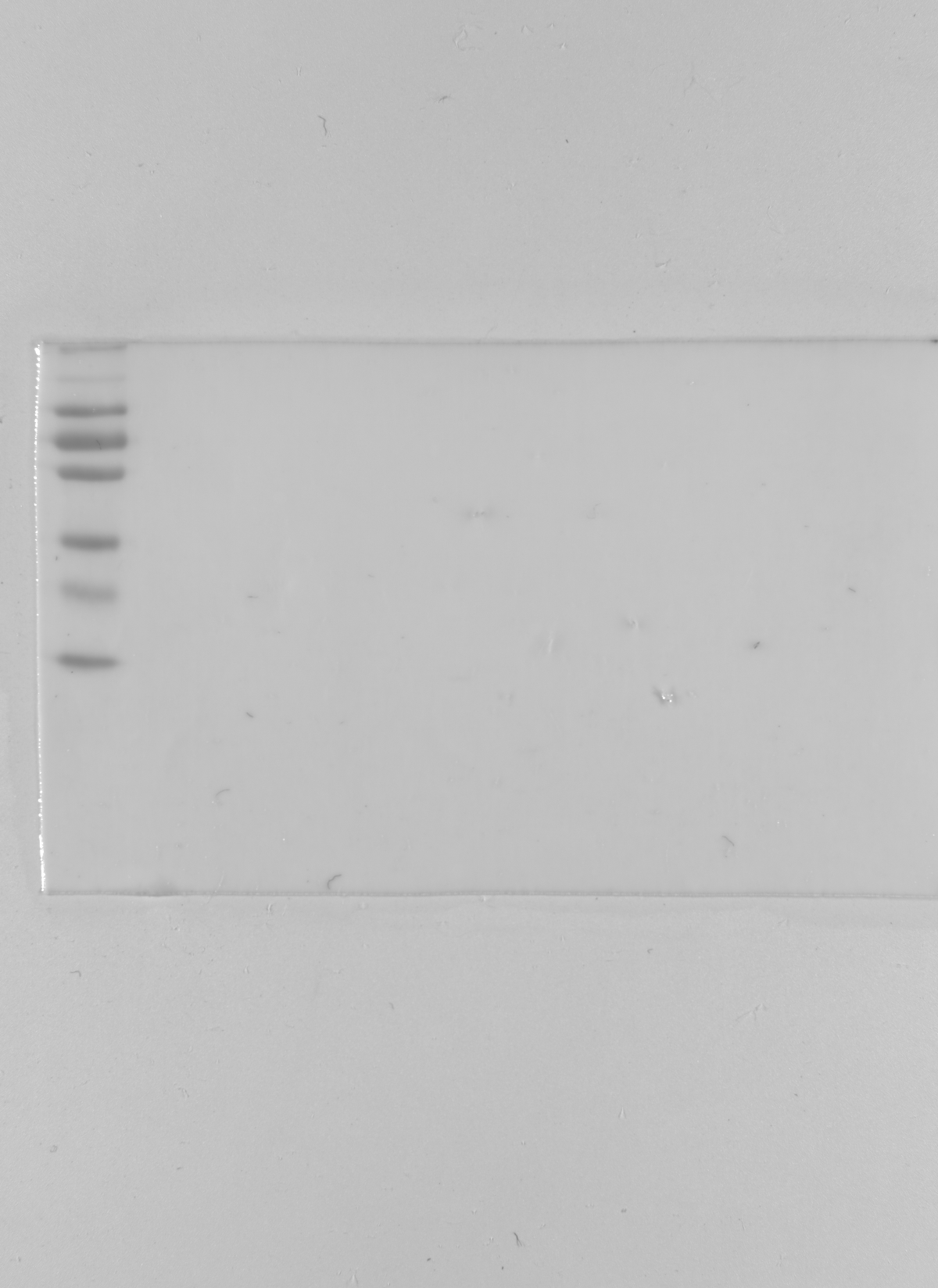

Supplement: Supplementary file 1 [file nutrients-17-02431-s001.zip › colon-WB/6.8 actin 20240608_162313_Ch/6.8 actin 20240608_162313_Ch-Marker.tif]

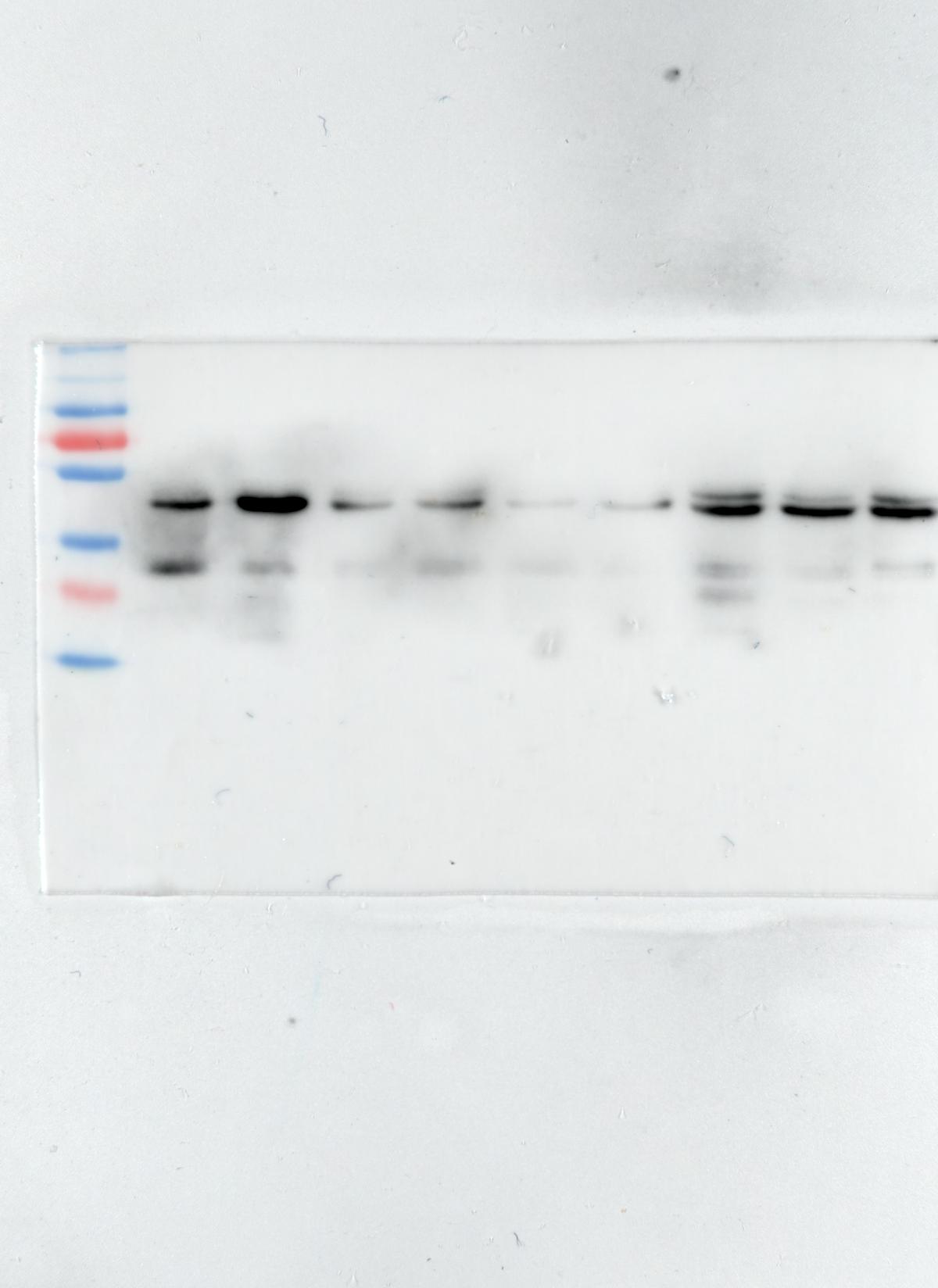

Supplement: Supplementary file 1 [file nutrients-17-02431-s001.zip › colon-WB/6.8 actin 20240608_162313_Ch/6.8 actin 20240608_162313_Ch_Chemi+Marker.jpg]

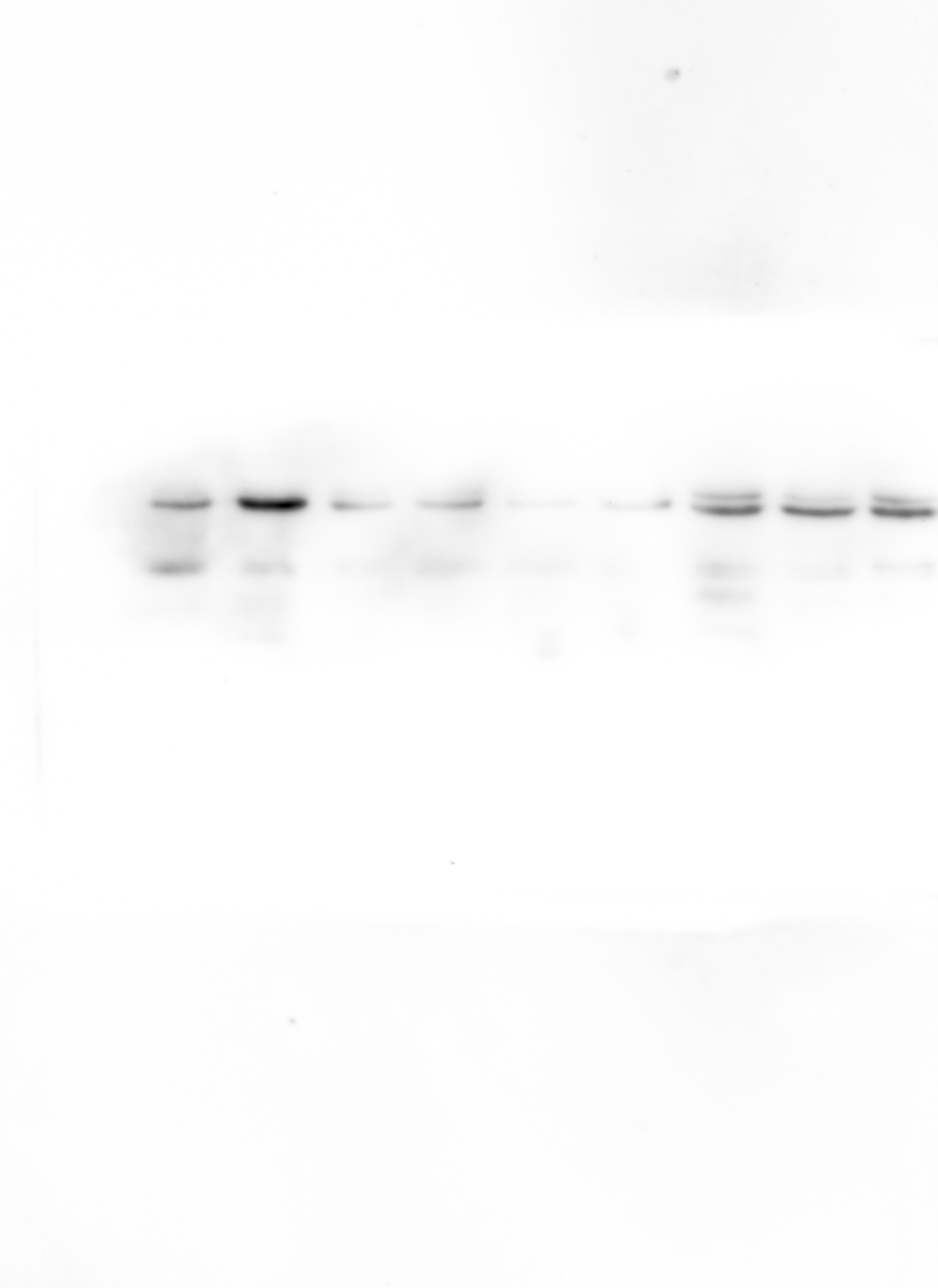

Supplement: Supplementary file 1 [file nutrients-17-02431-s001.zip › colon-WB/6.8 actin 20240608_162313_Ch/6.8 actin 20240608_162313_Ch_Chemi.tif]

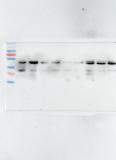

Supplement: Supplementary file 1 [file nutrients-17-02431-s001.zip › colon-WB/6.8 actin 20240608_162313_Ch/6.8 actin 20240608_162313_Ch_Thumb.jpg]

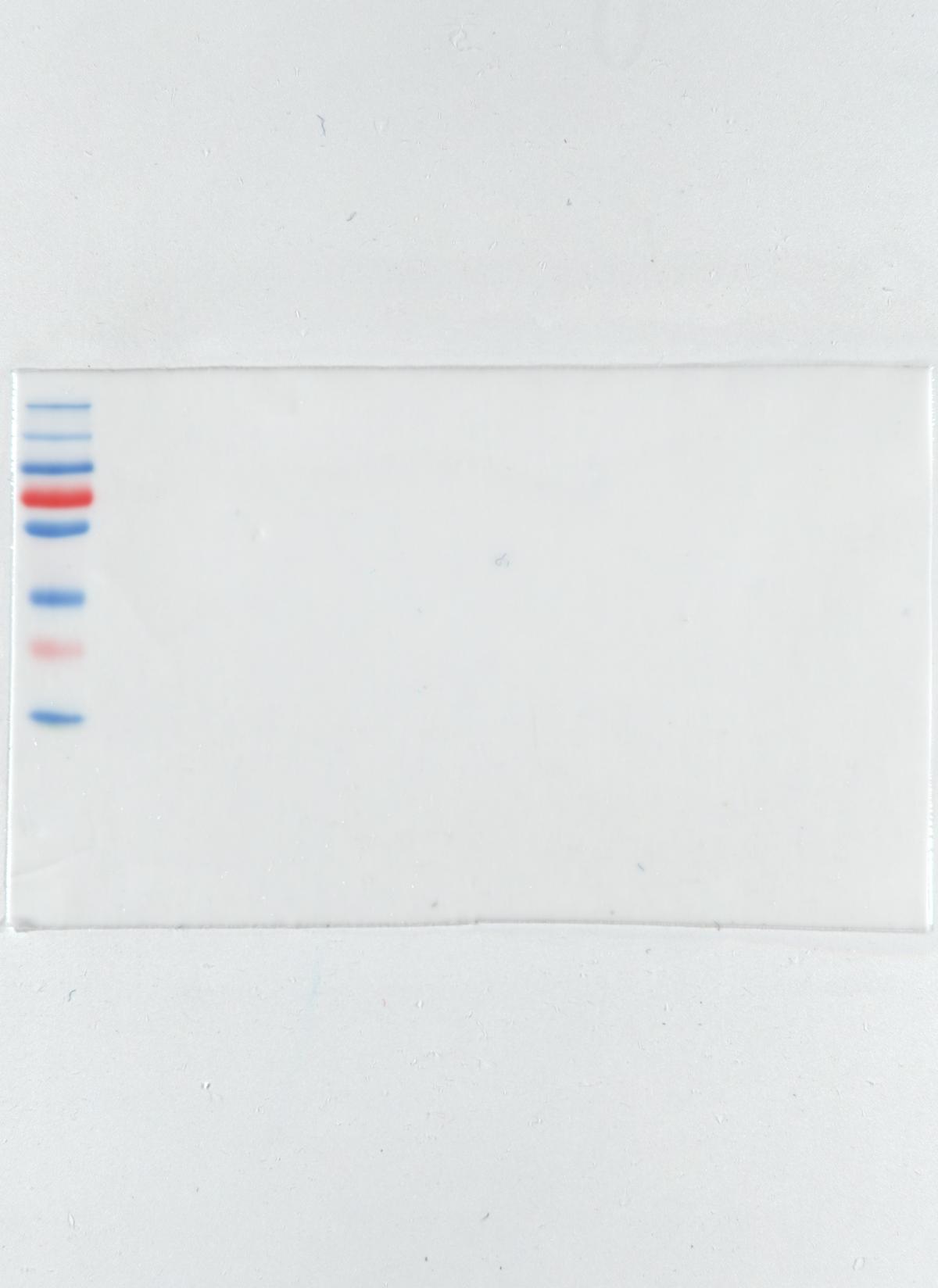

Supplement: Supplementary file 1 [file nutrients-17-02431-s001.zip › colon-WB/6.8 occludin 20240608_163314_Ch/6.8 occludin 20240608_163314_Ch-Marker.jpg]

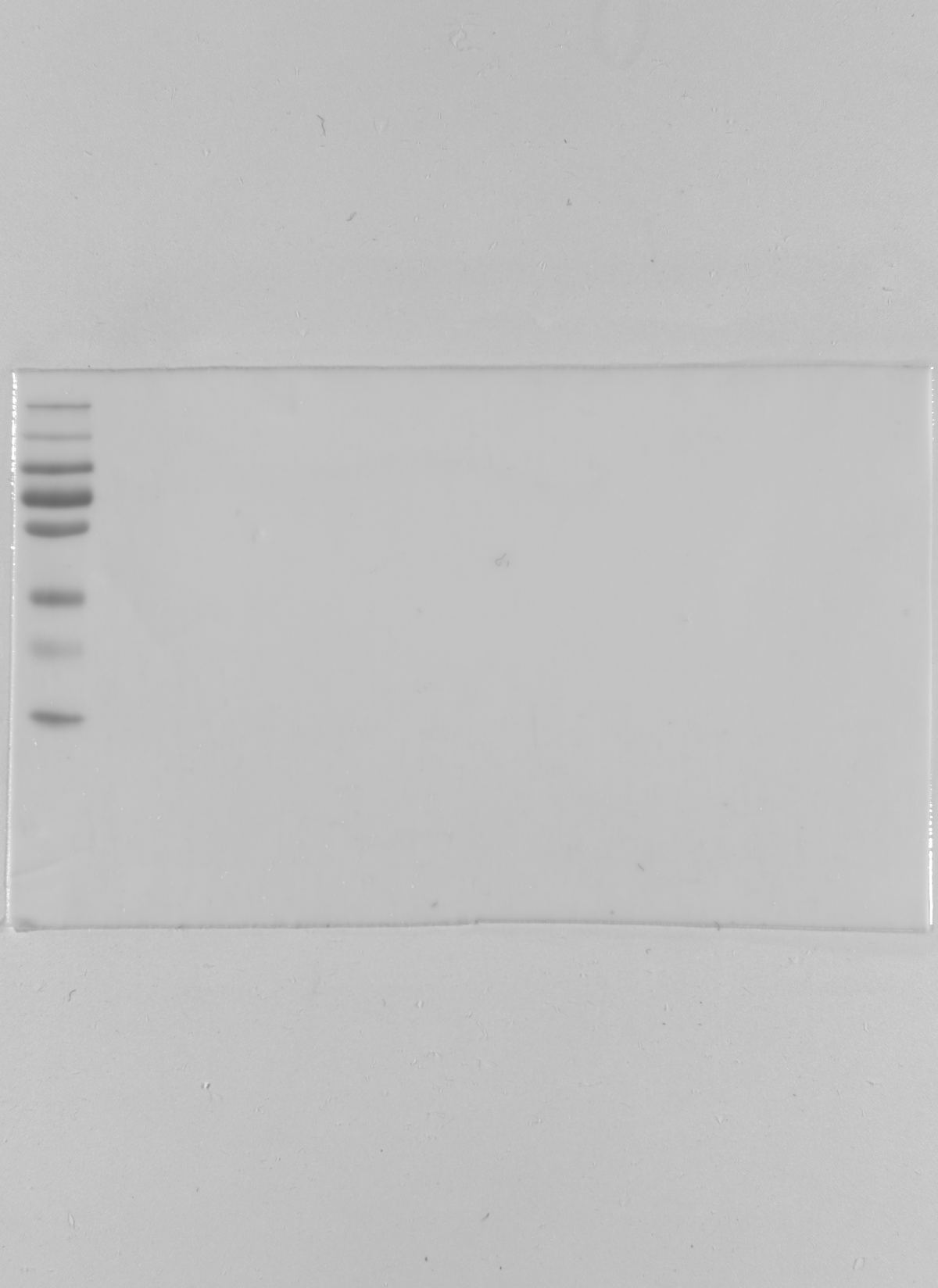

Supplement: Supplementary file 1 [file nutrients-17-02431-s001.zip › colon-WB/6.8 occludin 20240608_163314_Ch/6.8 occludin 20240608_163314_Ch-Marker.tif]

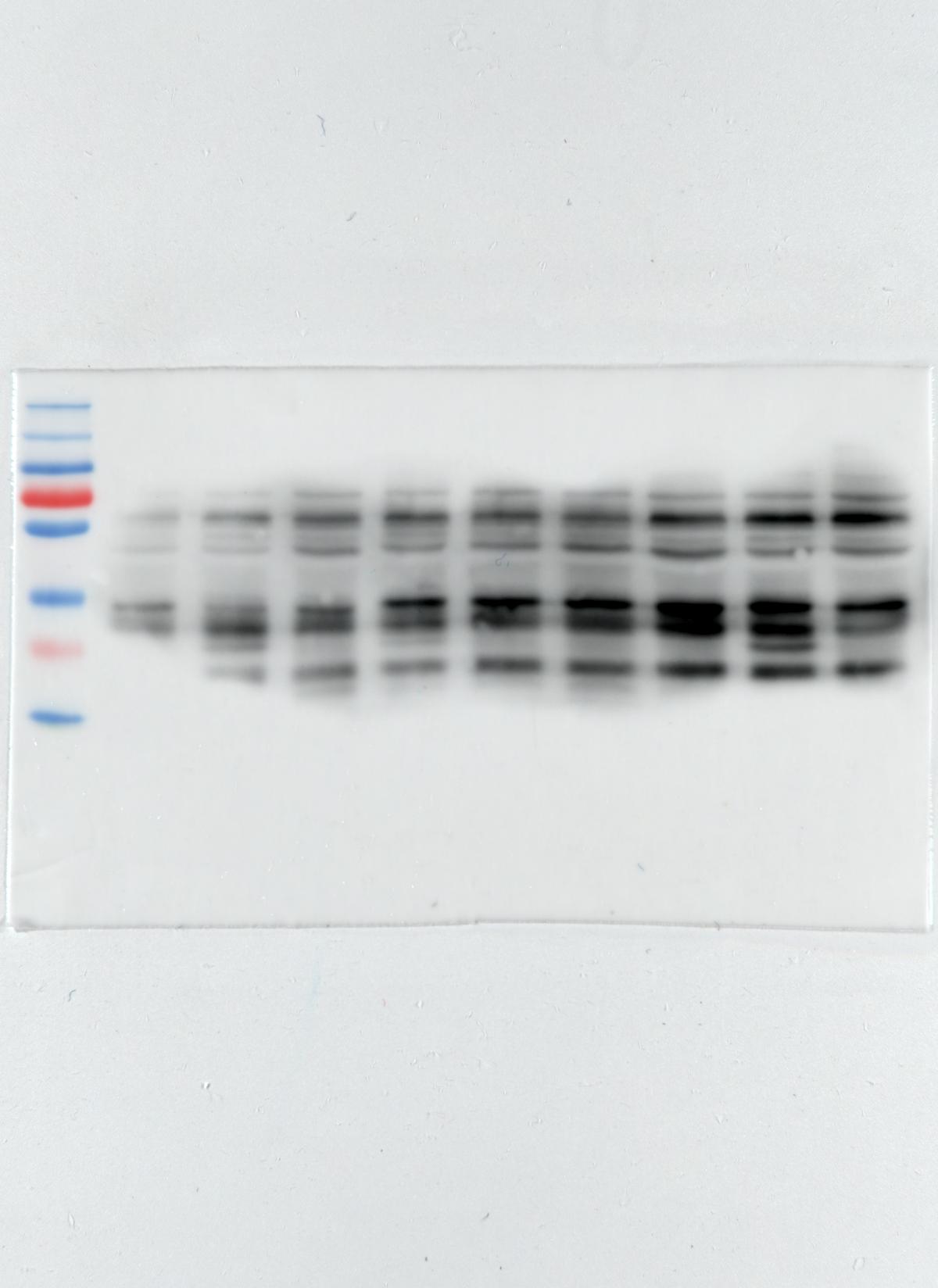

Supplement: Supplementary file 1 [file nutrients-17-02431-s001.zip › colon-WB/6.8 occludin 20240608_163314_Ch/6.8 occludin 20240608_163314_Ch_Chemi+Marker.jpg]

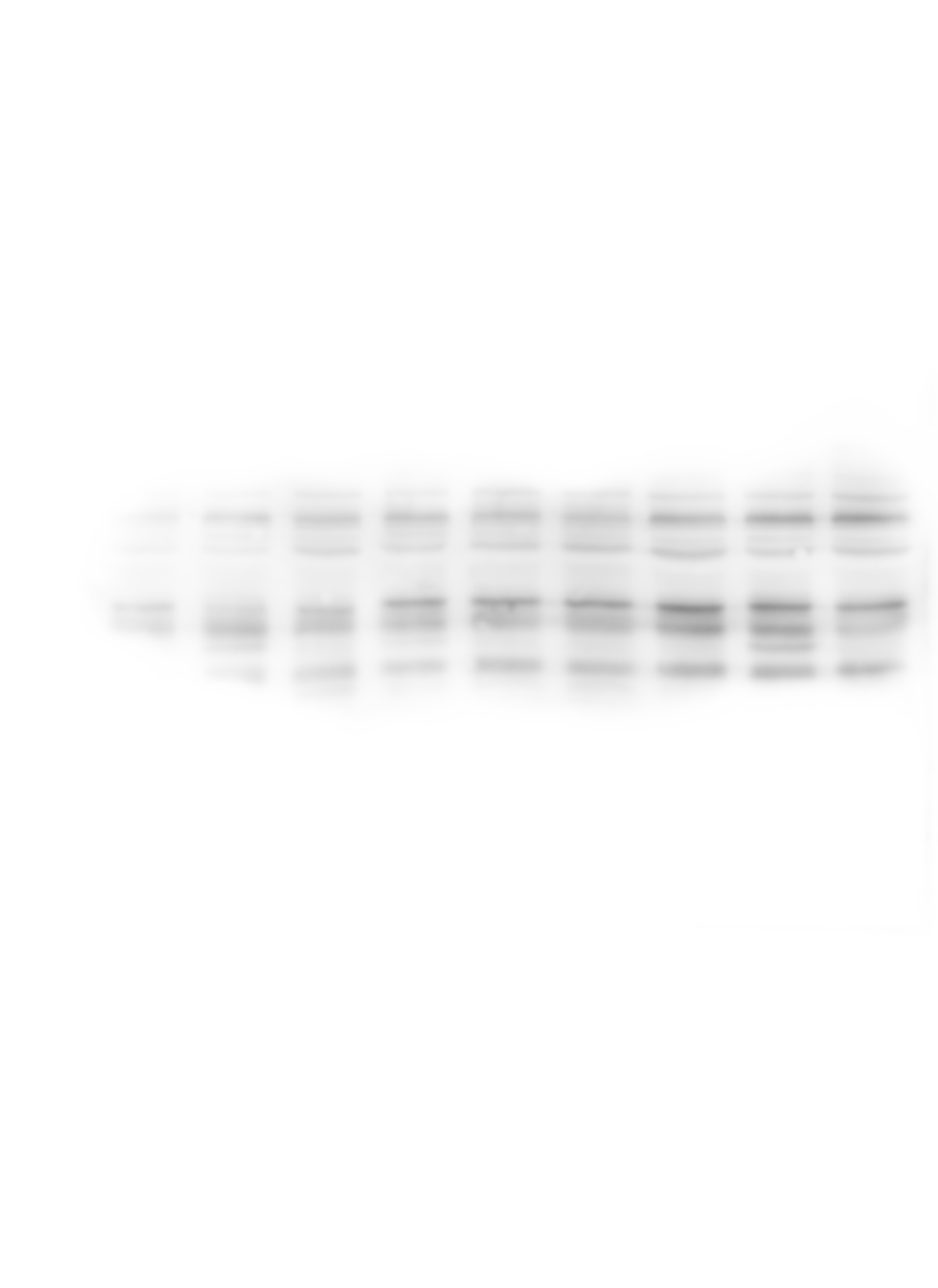

Supplement: Supplementary file 1 [file nutrients-17-02431-s001.zip › colon-WB/6.8 occludin 20240608_163314_Ch/6.8 occludin 20240608_163314_Ch_Chemi.tif]

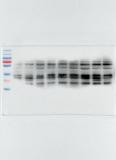

Supplement: Supplementary file 1 [file nutrients-17-02431-s001.zip › colon-WB/6.8 occludin 20240608_163314_Ch/6.8 occludin 20240608_163314_Ch_Thumb.jpg]

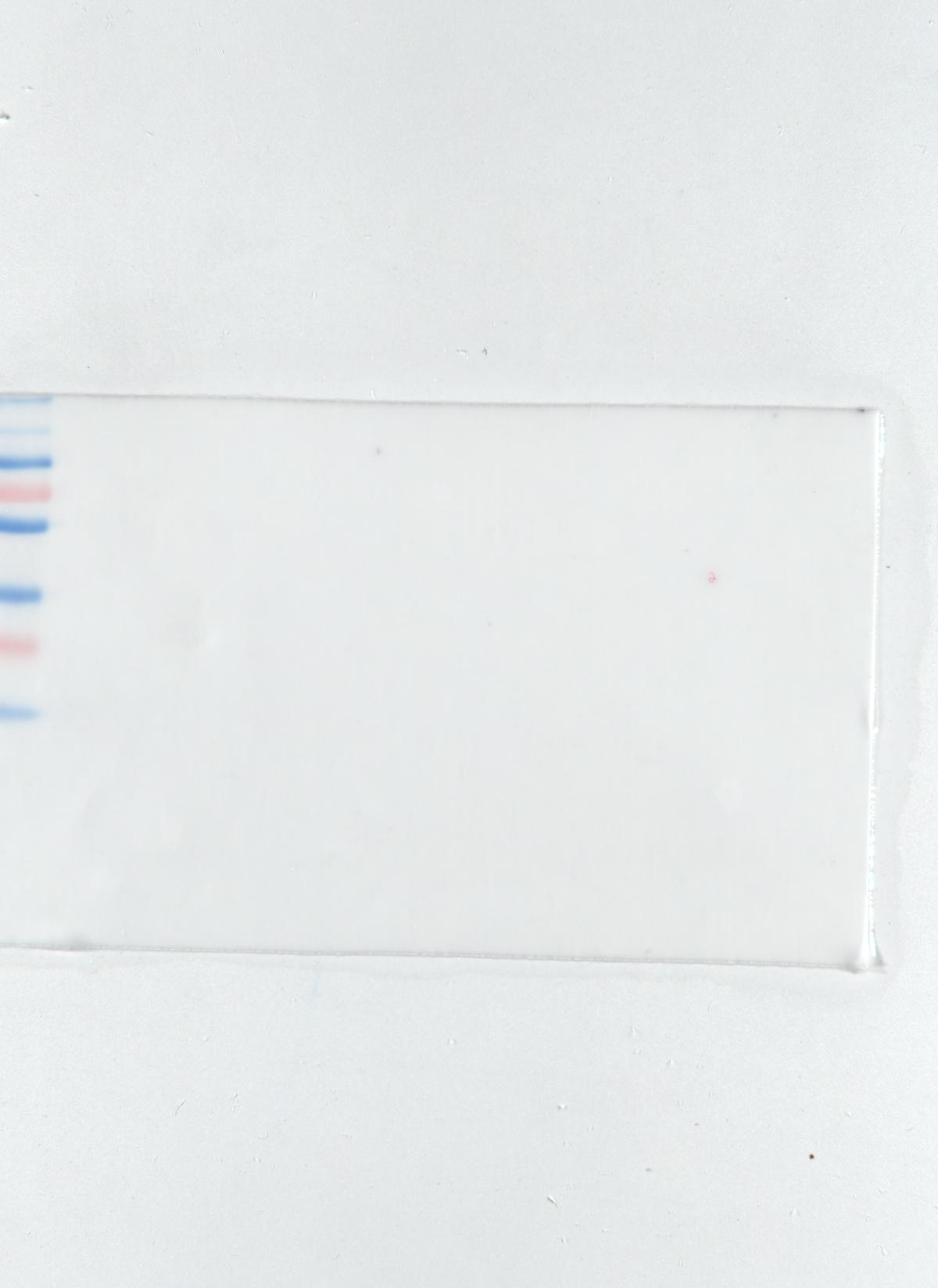

Supplement: Supplementary file 1 [file nutrients-17-02431-s001.zip › colon-WB/6.9 actin 20240609_160124_Ch/actin 20240609_160124_Ch-Marker.jpg]

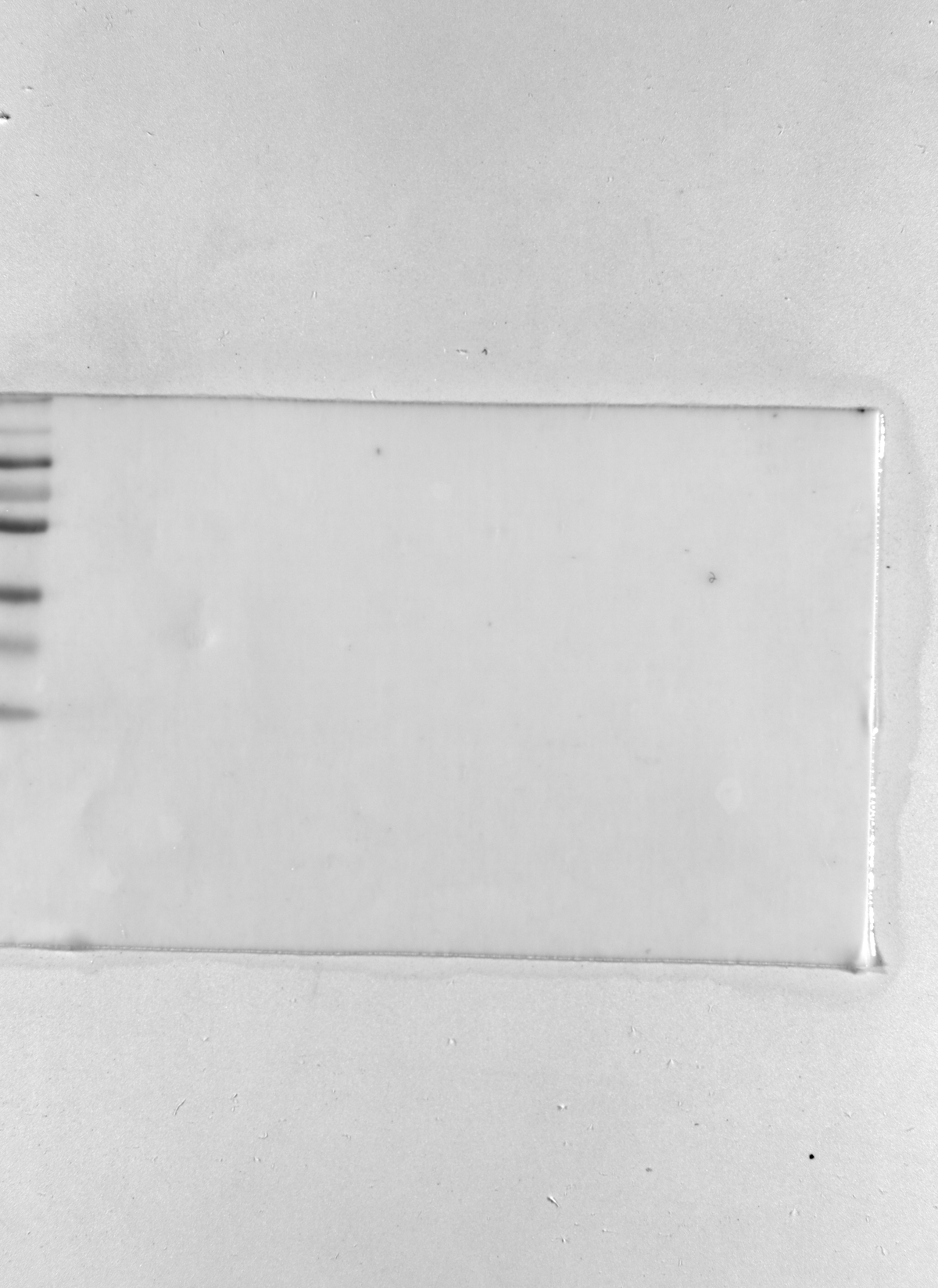

Supplement: Supplementary file 1 [file nutrients-17-02431-s001.zip › colon-WB/6.9 actin 20240609_160124_Ch/actin 20240609_160124_Ch-Marker.tif]

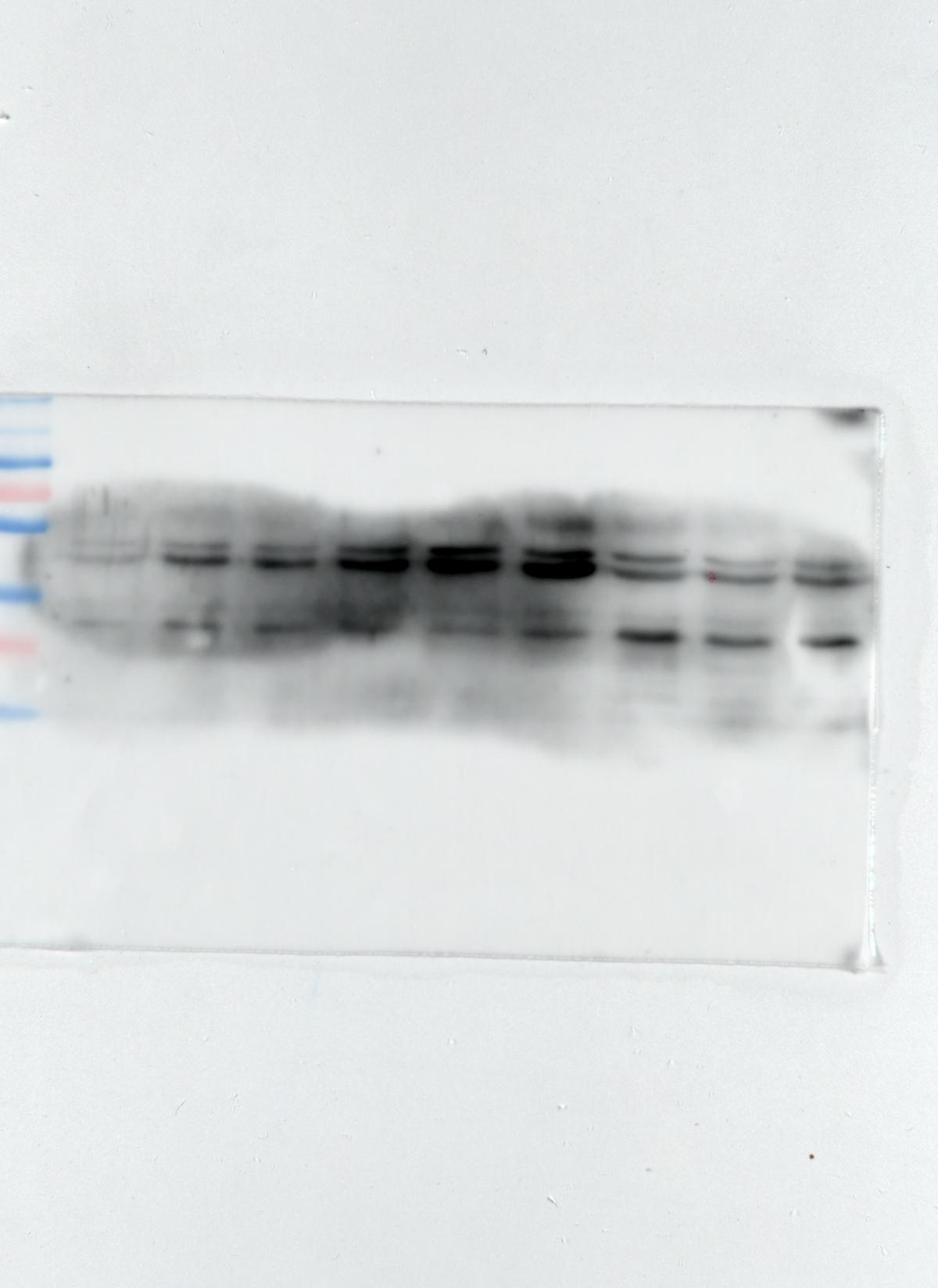

Supplement: Supplementary file 1 [file nutrients-17-02431-s001.zip › colon-WB/6.9 actin 20240609_160124_Ch/actin 20240609_160124_Ch_Chemi+Marker.jpg]

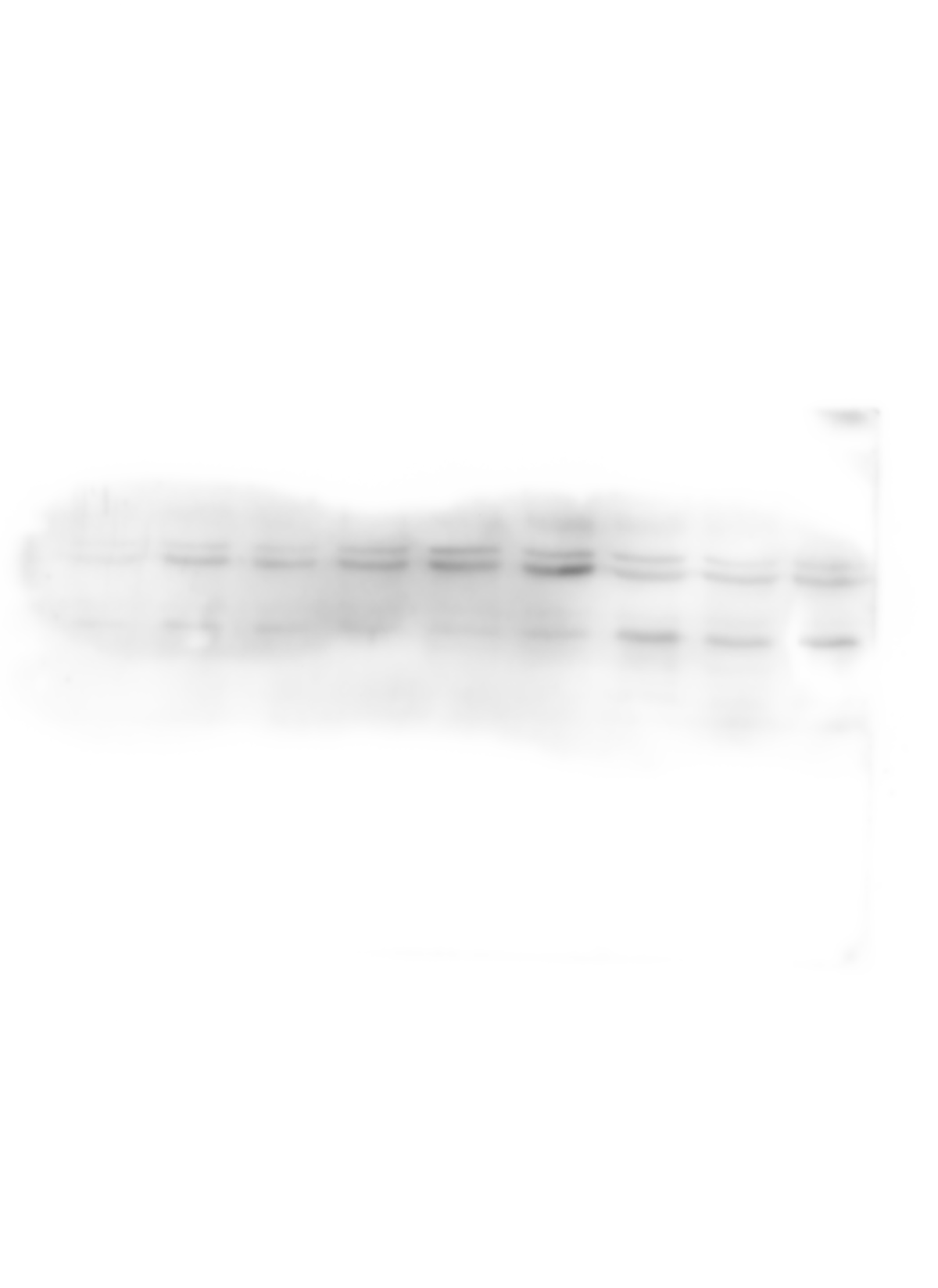

Supplement: Supplementary file 1 [file nutrients-17-02431-s001.zip › colon-WB/6.9 actin 20240609_160124_Ch/actin 20240609_160124_Ch_Chemi.tif]

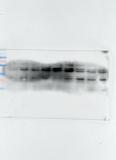

Supplement: Supplementary file 1 [file nutrients-17-02431-s001.zip › colon-WB/6.9 actin 20240609_160124_Ch/actin 20240609_160124_Ch_Thumb.jpg]

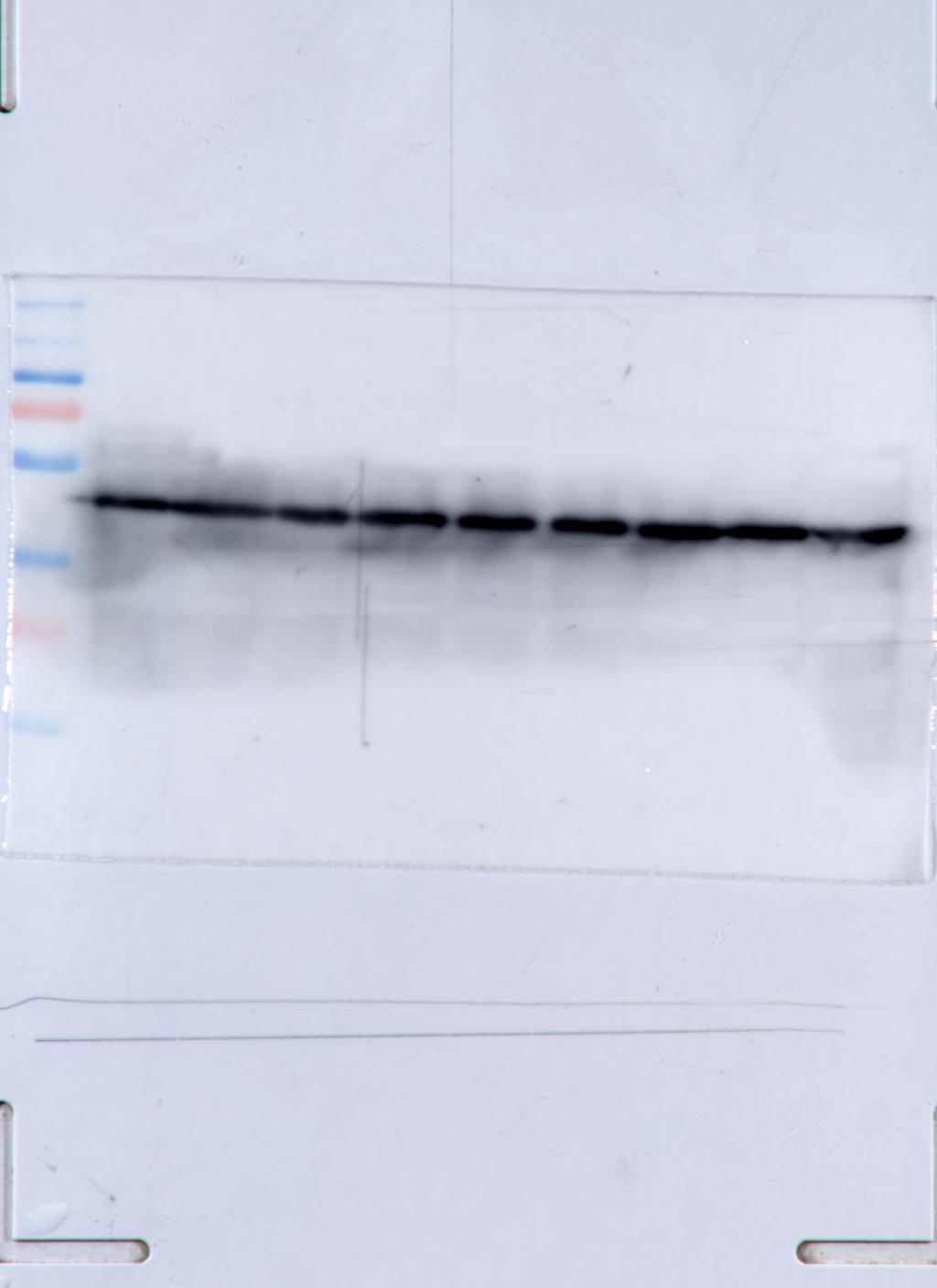

Supplement: Supplementary file 1 [file nutrients-17-02431-s001.zip › colon-WB/7.2actin 2024.07.02_13.15.26_Ch/7.2actin 2024.07.02_13.15.26_Ch+Marker.jpg]

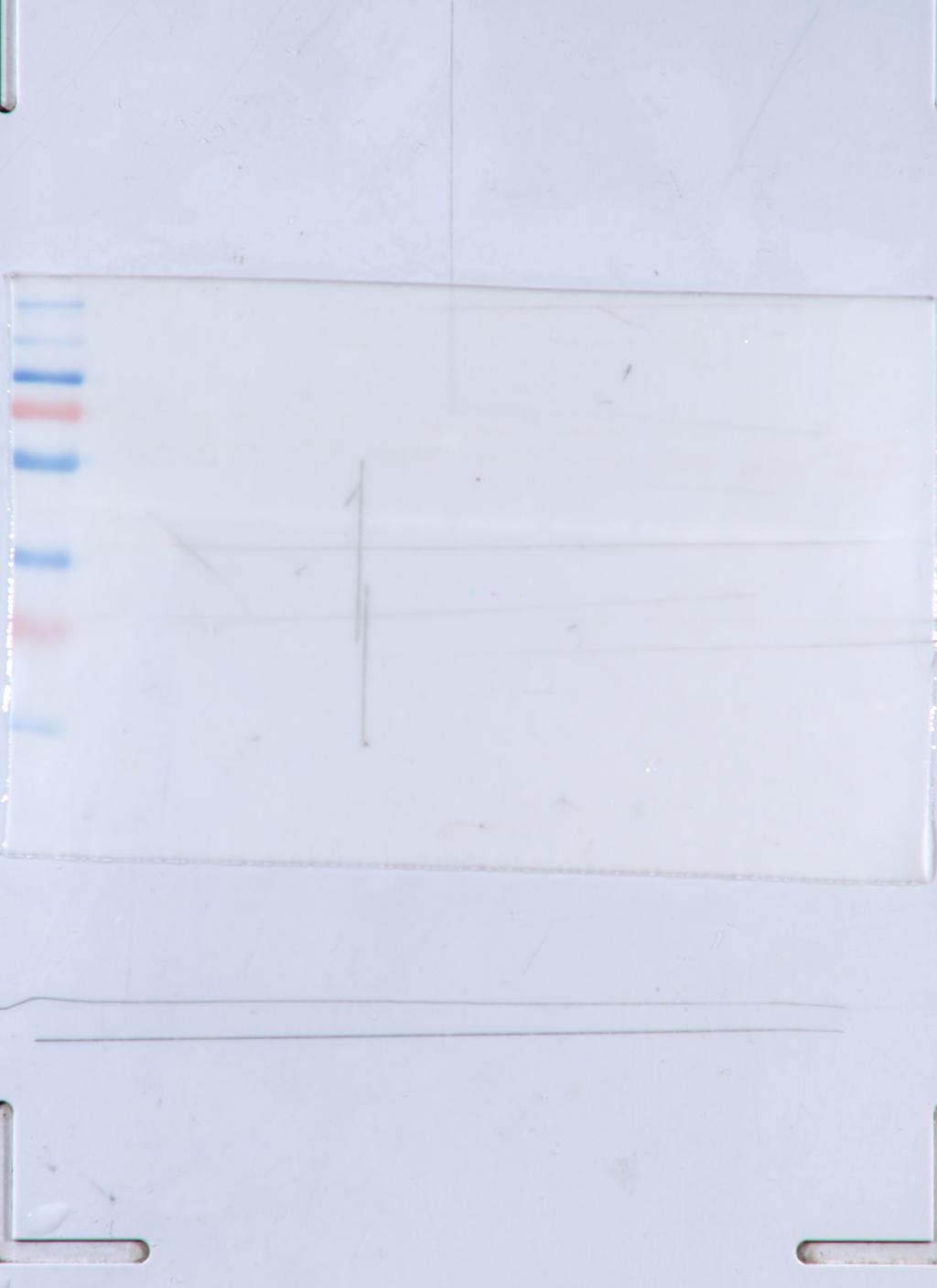

Supplement: Supplementary file 1 [file nutrients-17-02431-s001.zip › colon-WB/7.2actin 2024.07.02_13.15.26_Ch/7.2actin 2024.07.02_13.15.26_Ch-Marker.jpg]

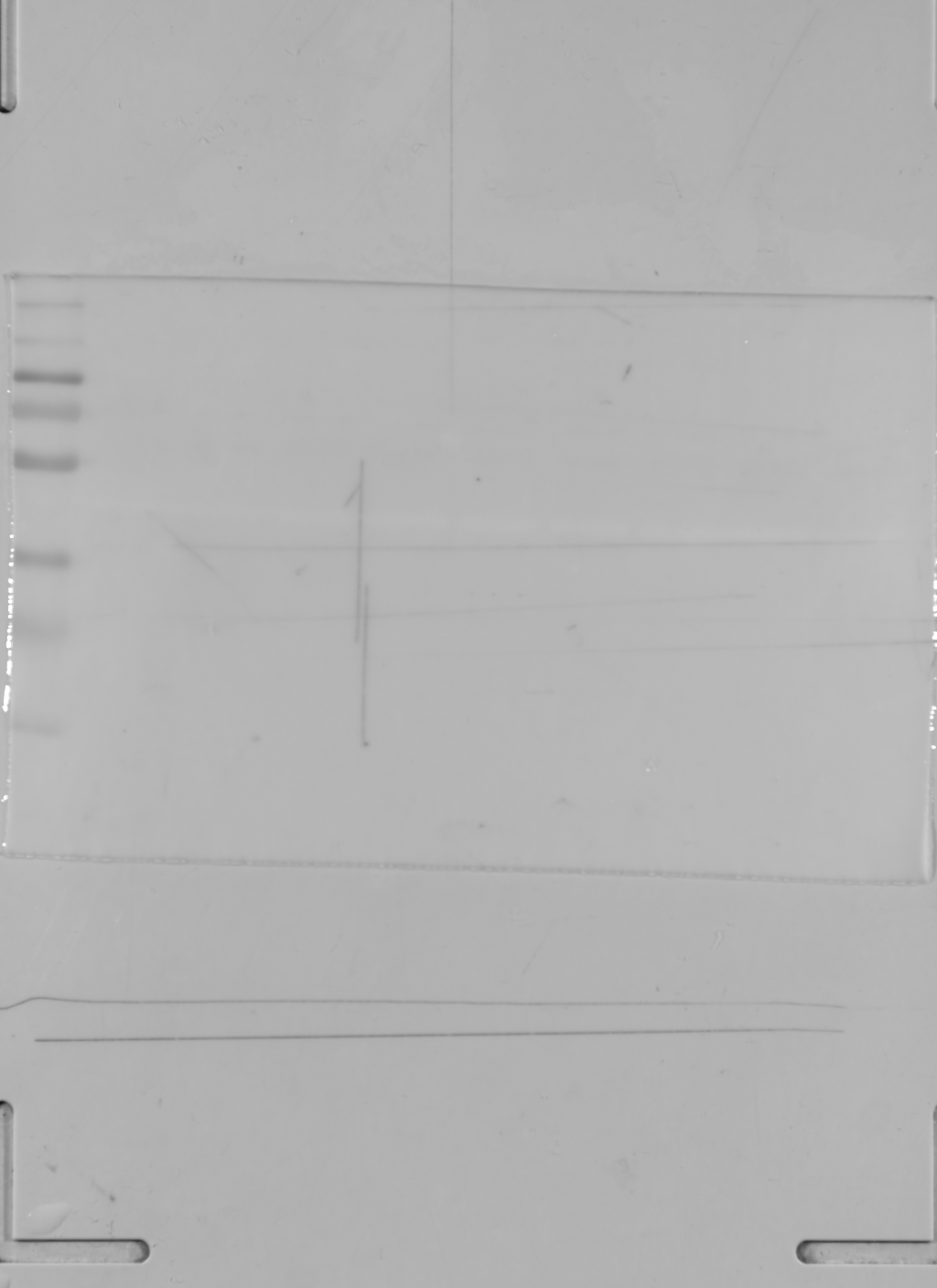

Supplement: Supplementary file 1 [file nutrients-17-02431-s001.zip › colon-WB/7.2actin 2024.07.02_13.15.26_Ch/7.2actin 2024.07.02_13.15.26_Ch-Marker.tif]

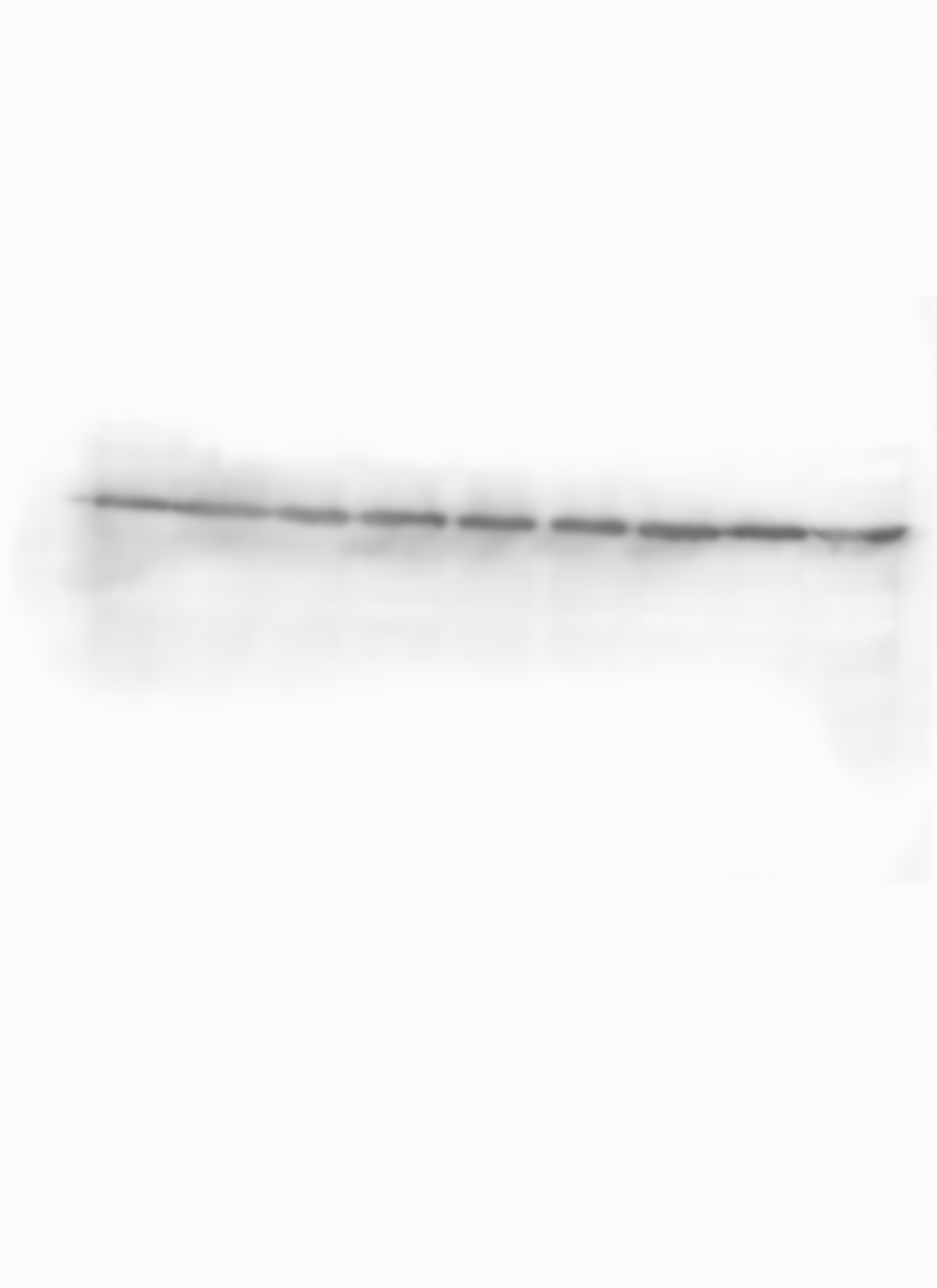

Supplement: Supplementary file 1 [file nutrients-17-02431-s001.zip › colon-WB/7.2actin 2024.07.02_13.15.26_Ch/7.2actin 2024.07.02_13.15.26_Ch.tif]

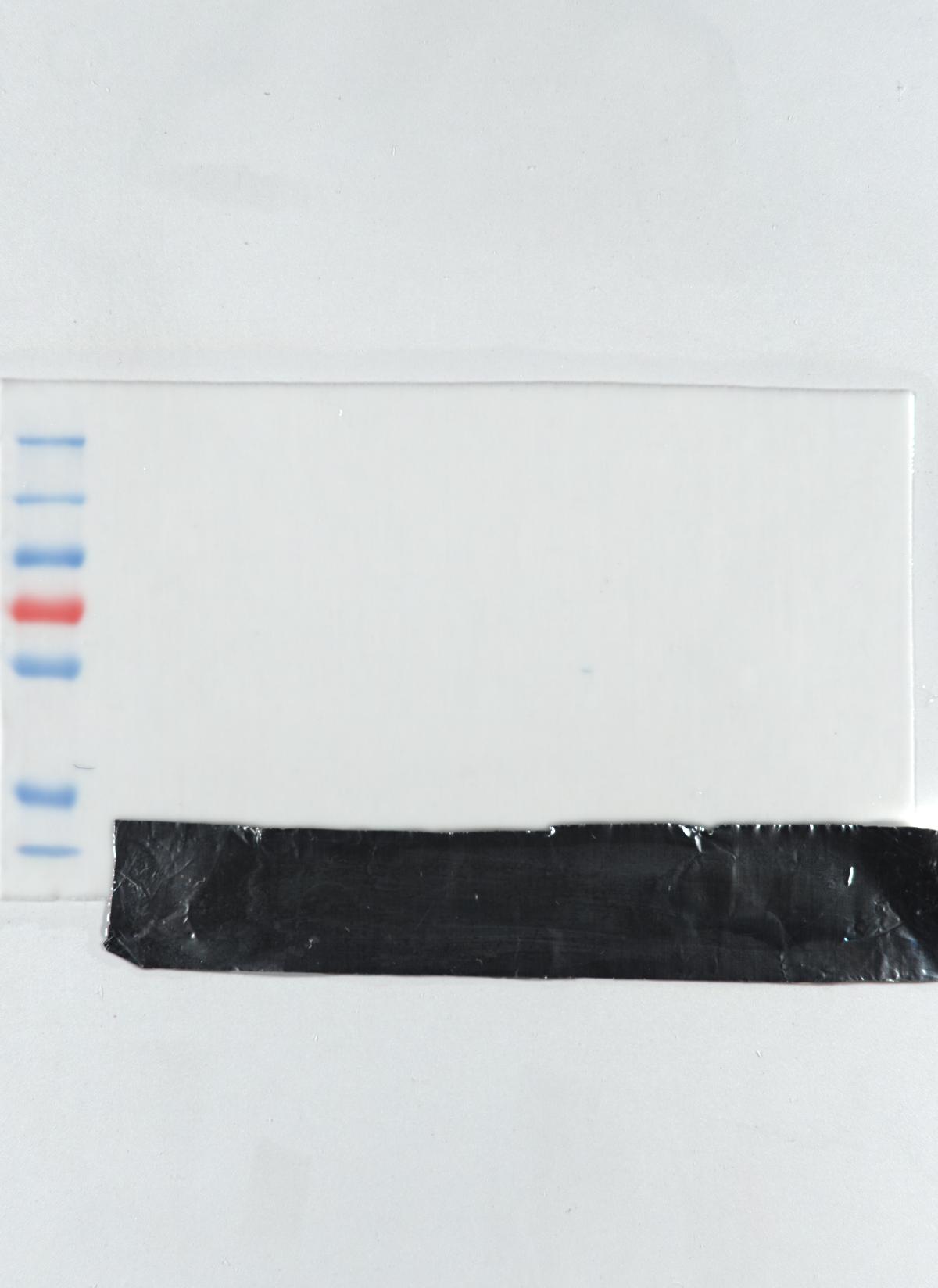

Supplement: Supplementary file 1 [file nutrients-17-02431-s001.zip › colon-WB/7.4 actin 20240704_004001_Ch/7.4 actin 20240704_004001_Ch-Marker.jpg]

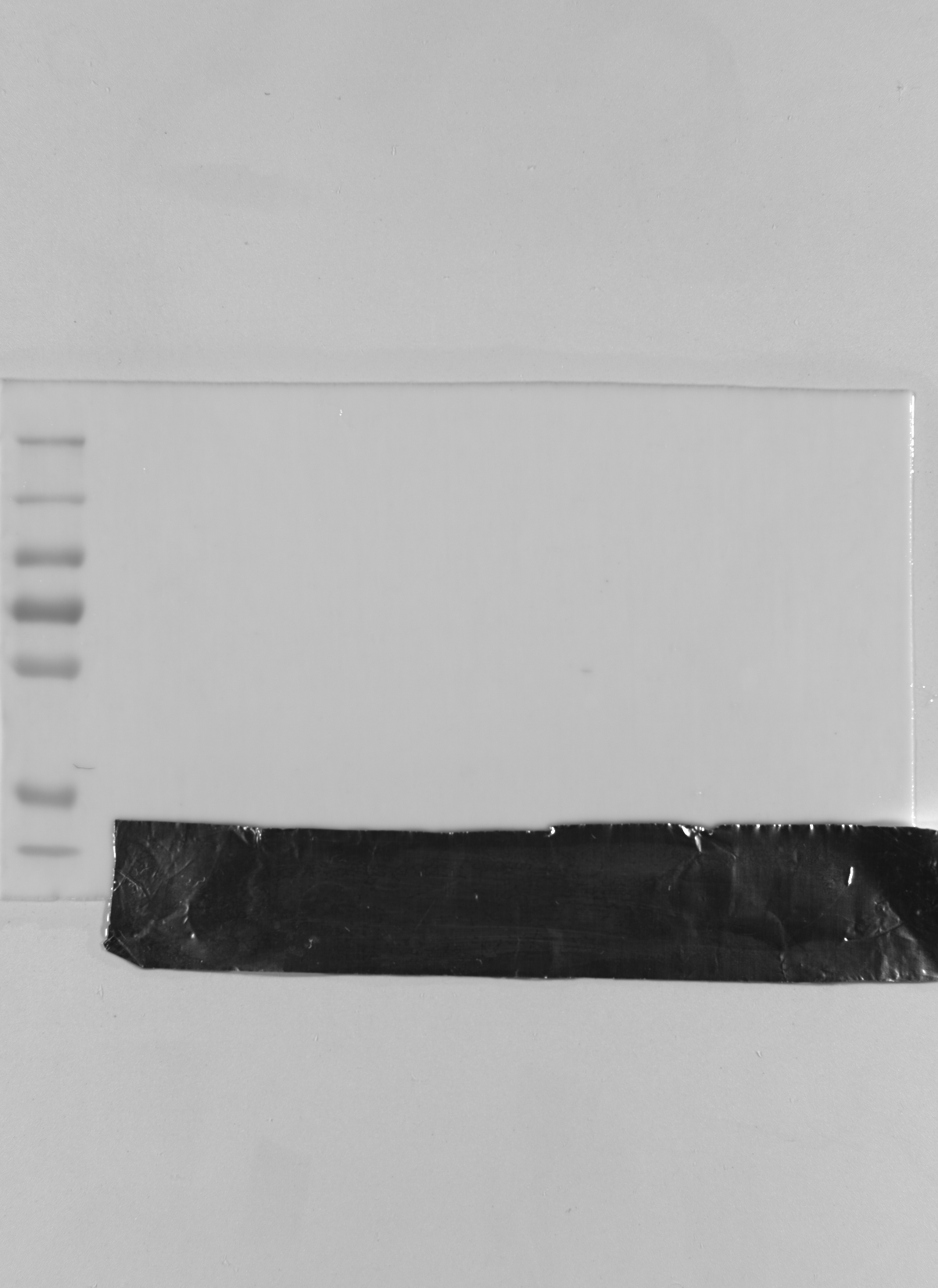

Supplement: Supplementary file 1 [file nutrients-17-02431-s001.zip › colon-WB/7.4 actin 20240704_004001_Ch/7.4 actin 20240704_004001_Ch-Marker.tif]

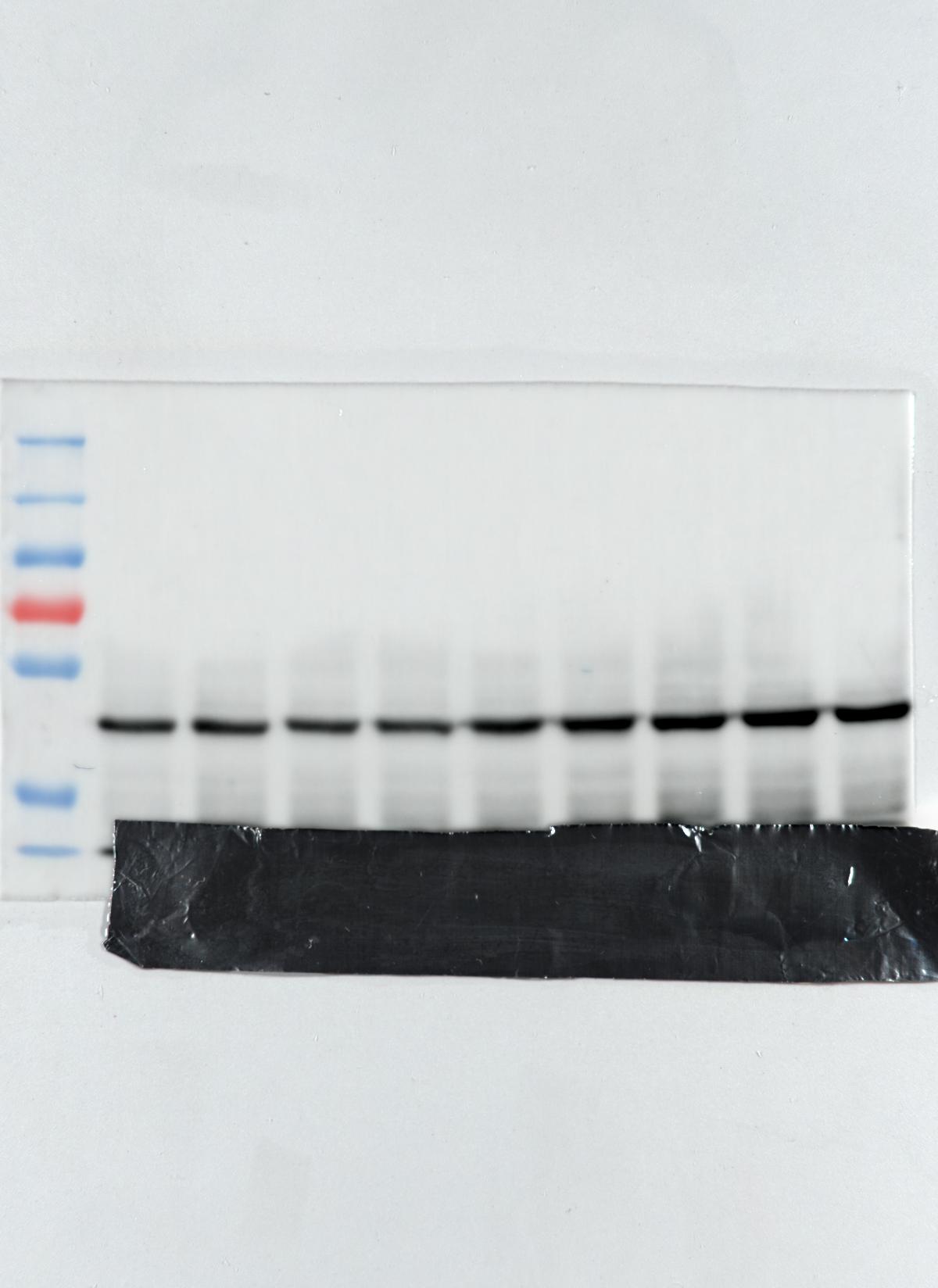

Supplement: Supplementary file 1 [file nutrients-17-02431-s001.zip › colon-WB/7.4 actin 20240704_004001_Ch/7.4 actin 20240704_004001_Ch_Chemi+Marker.jpg]

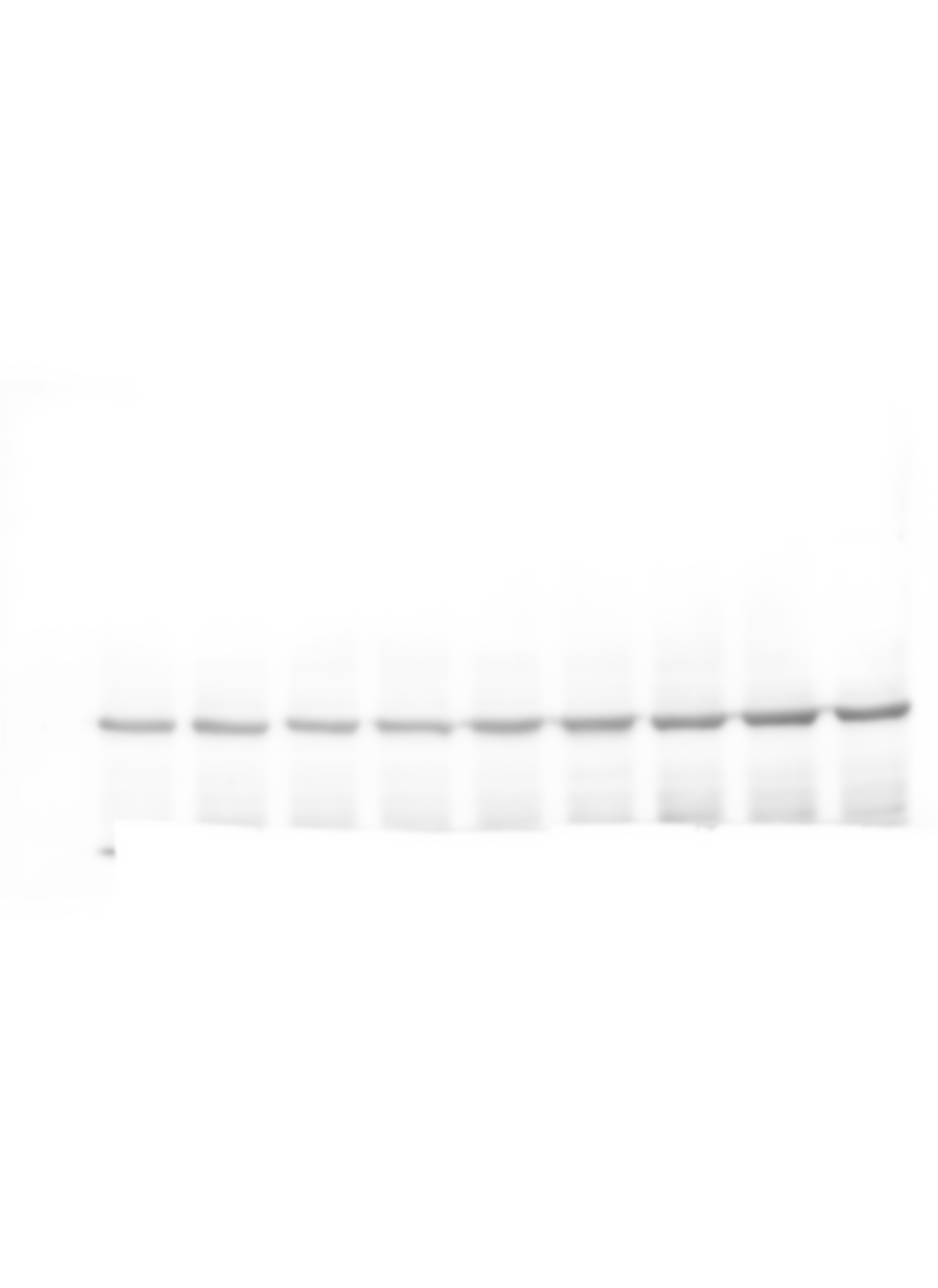

Supplement: Supplementary file 1 [file nutrients-17-02431-s001.zip › colon-WB/7.4 actin 20240704_004001_Ch/7.4 actin 20240704_004001_Ch_Chemi.tif]

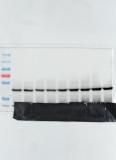

Supplement: Supplementary file 1 [file nutrients-17-02431-s001.zip › colon-WB/7.4 actin 20240704_004001_Ch/7.4 actin 20240704_004001_Ch_Thumb.jpg]

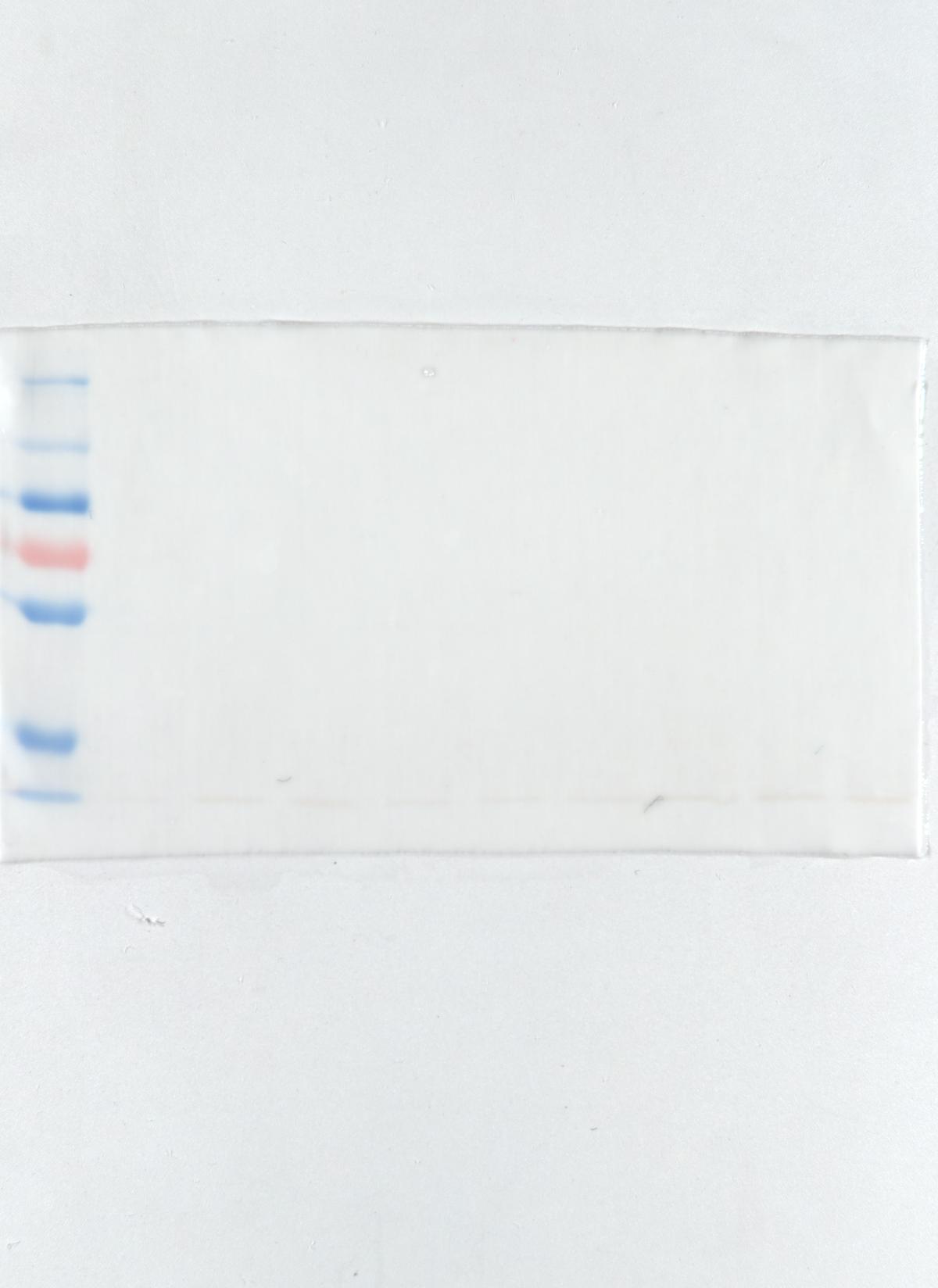

Supplement: Supplementary file 1 [file nutrients-17-02431-s001.zip › colon-WB/7.4 occludin 20240709_102122_Ch/7.4 occludin 20240709_102122_Ch-Marker.jpg]

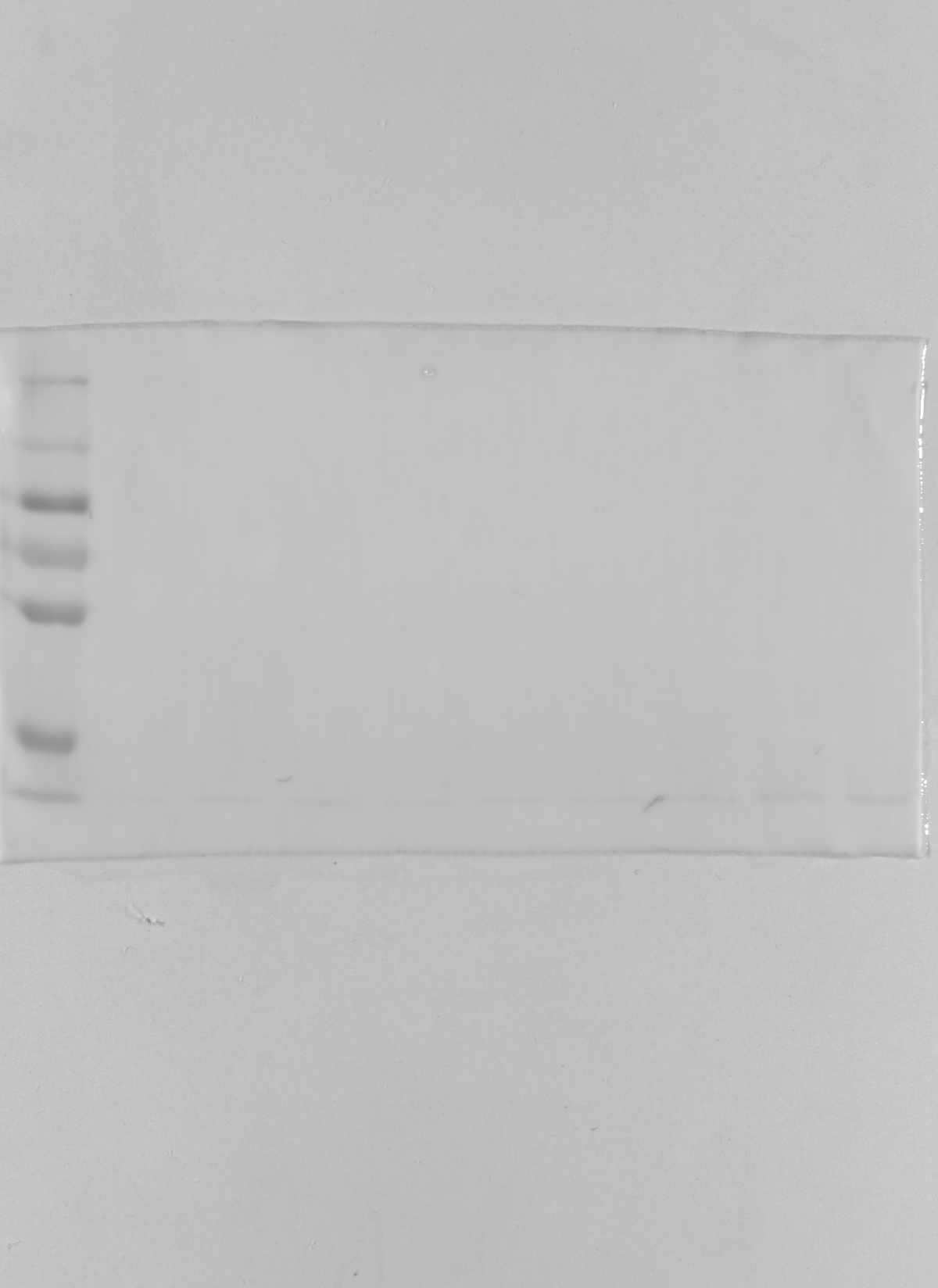

Supplement: Supplementary file 1 [file nutrients-17-02431-s001.zip › colon-WB/7.4 occludin 20240709_102122_Ch/7.4 occludin 20240709_102122_Ch-Marker.tif]

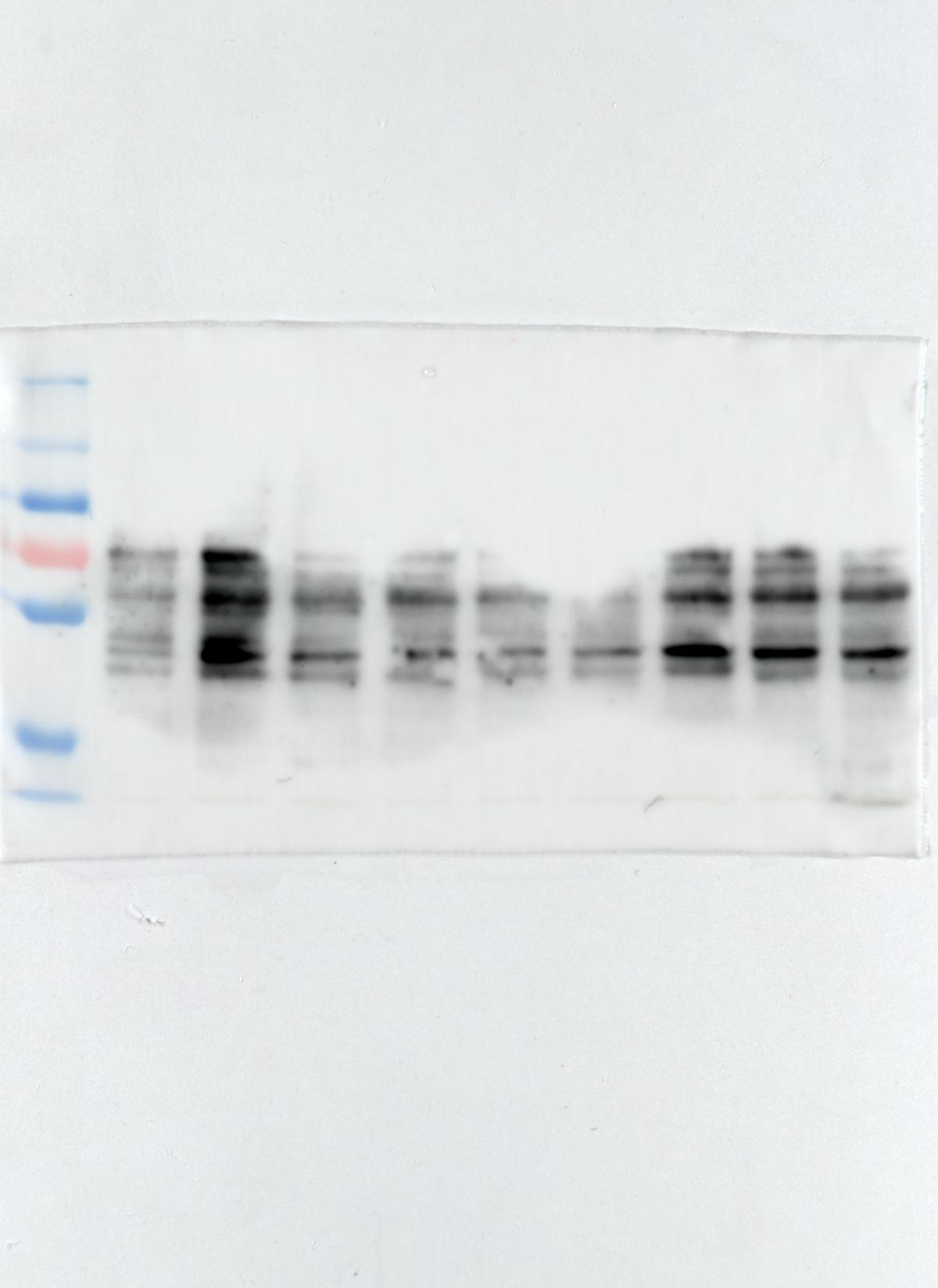

Supplement: Supplementary file 1 [file nutrients-17-02431-s001.zip › colon-WB/7.4 occludin 20240709_102122_Ch/7.4 occludin 20240709_102122_Ch_Chemi+Marker.jpg]

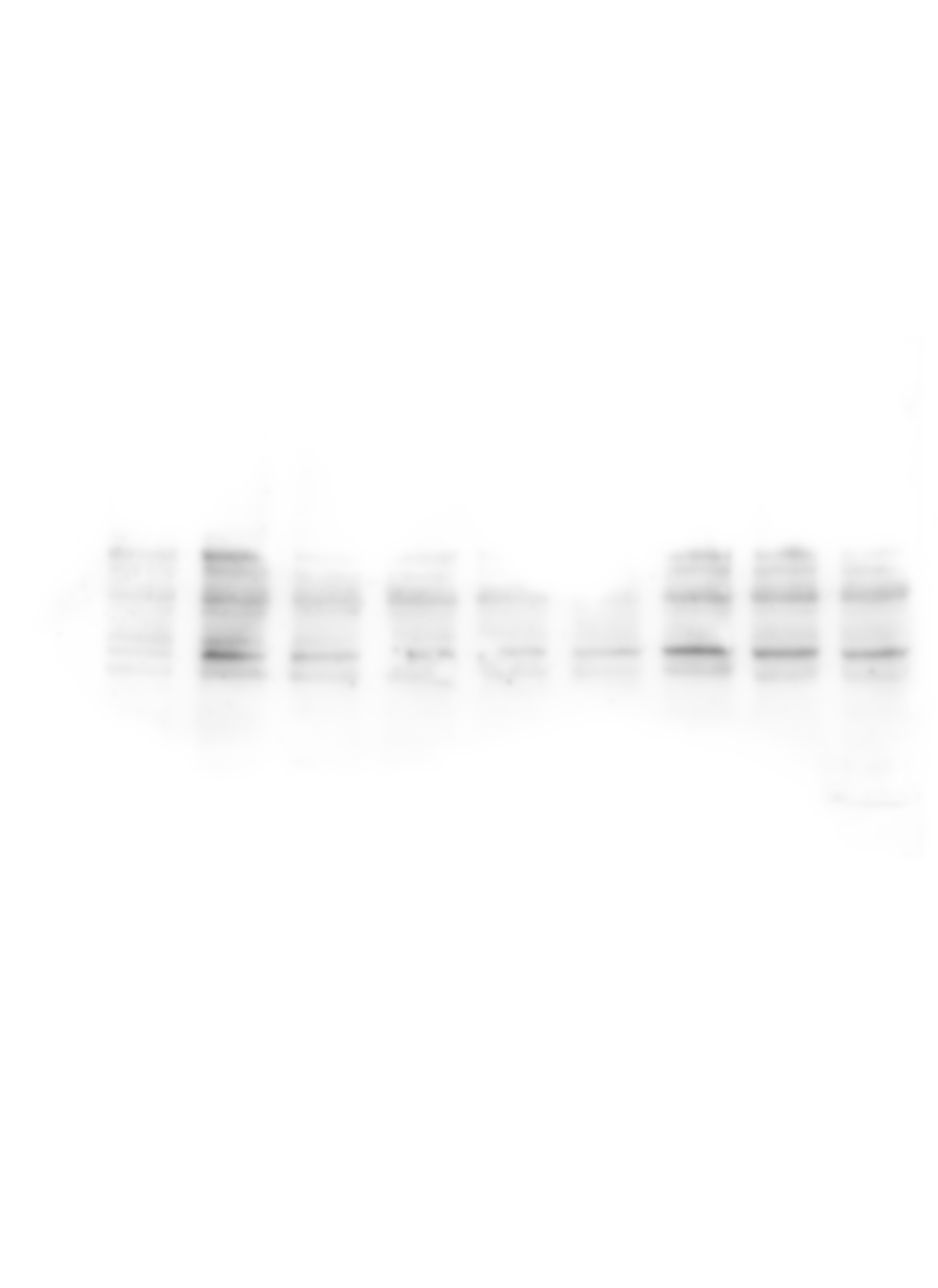

Supplement: Supplementary file 1 [file nutrients-17-02431-s001.zip › colon-WB/7.4 occludin 20240709_102122_Ch/7.4 occludin 20240709_102122_Ch_Chemi.tif]

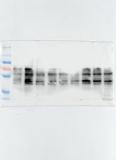

Supplement: Supplementary file 1 [file nutrients-17-02431-s001.zip › colon-WB/7.4 occludin 20240709_102122_Ch/7.4 occludin 20240709_102122_Ch_Thumb.jpg]

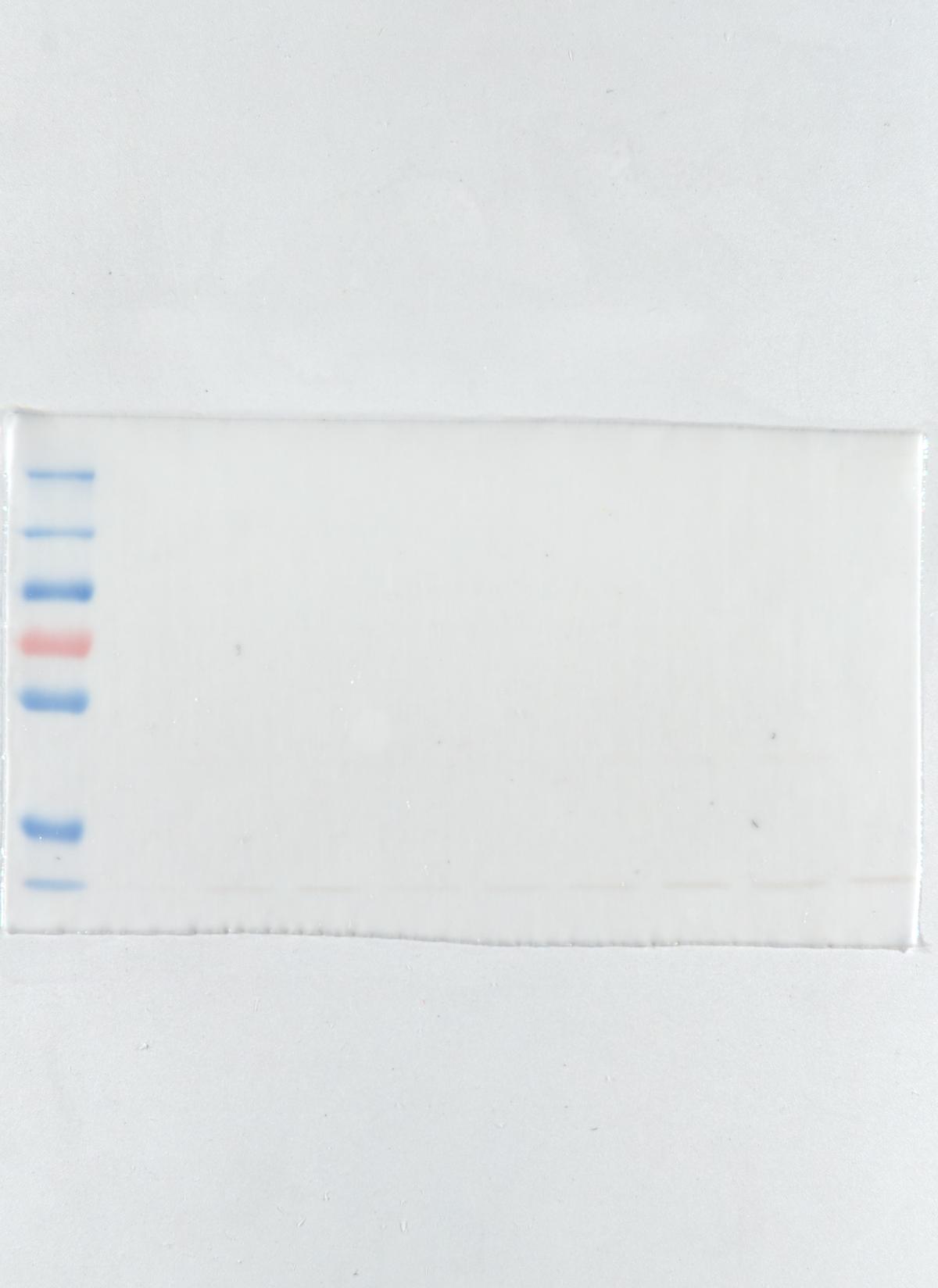

Supplement: Supplementary file 1 [file nutrients-17-02431-s001.zip › colon-WB/7.4 zo1 20240709_102519_Ch/7.4 zo1 20240709_102519_Ch-Marker.jpg]

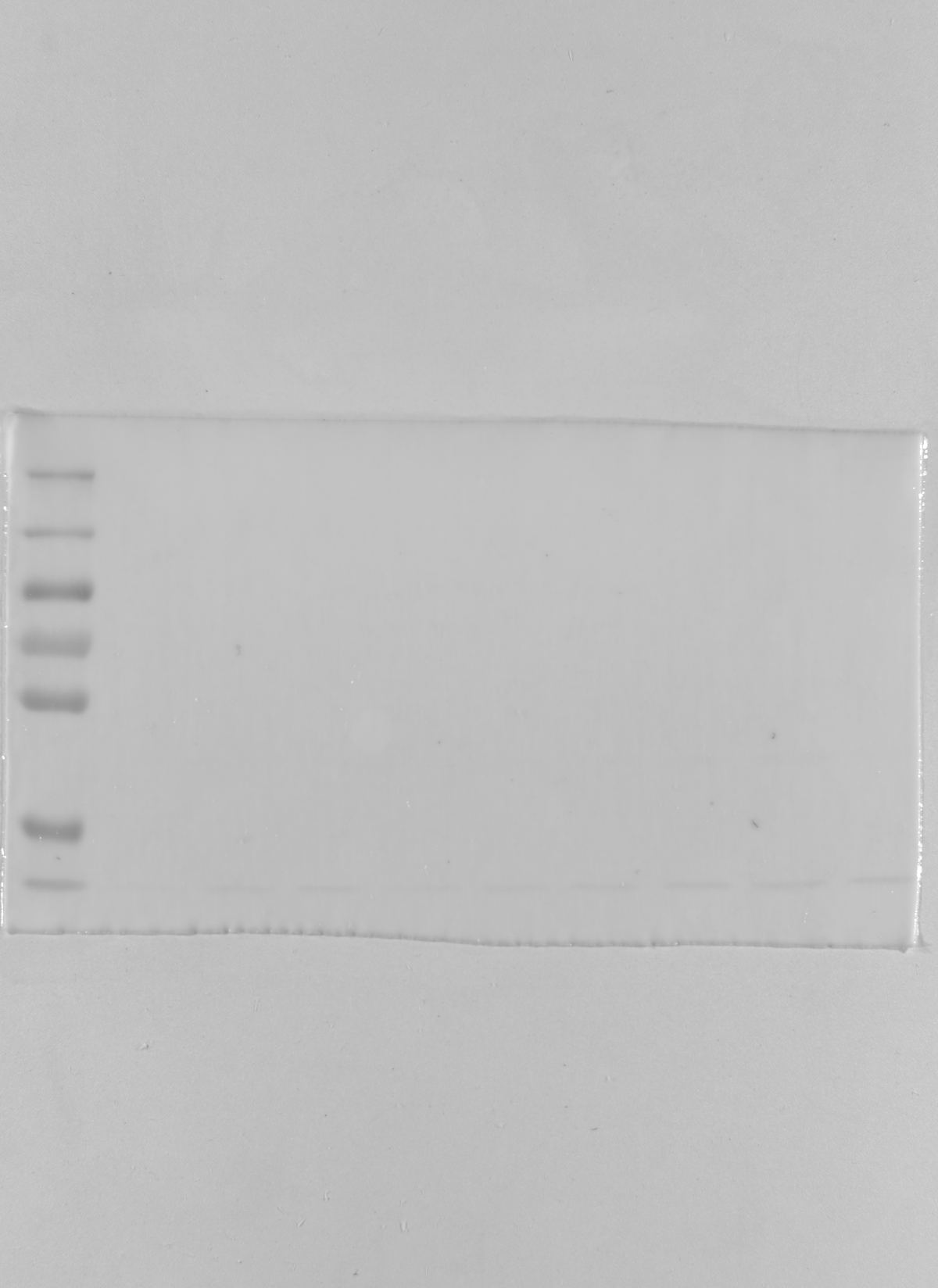

Supplement: Supplementary file 1 [file nutrients-17-02431-s001.zip › colon-WB/7.4 zo1 20240709_102519_Ch/7.4 zo1 20240709_102519_Ch-Marker.tif]

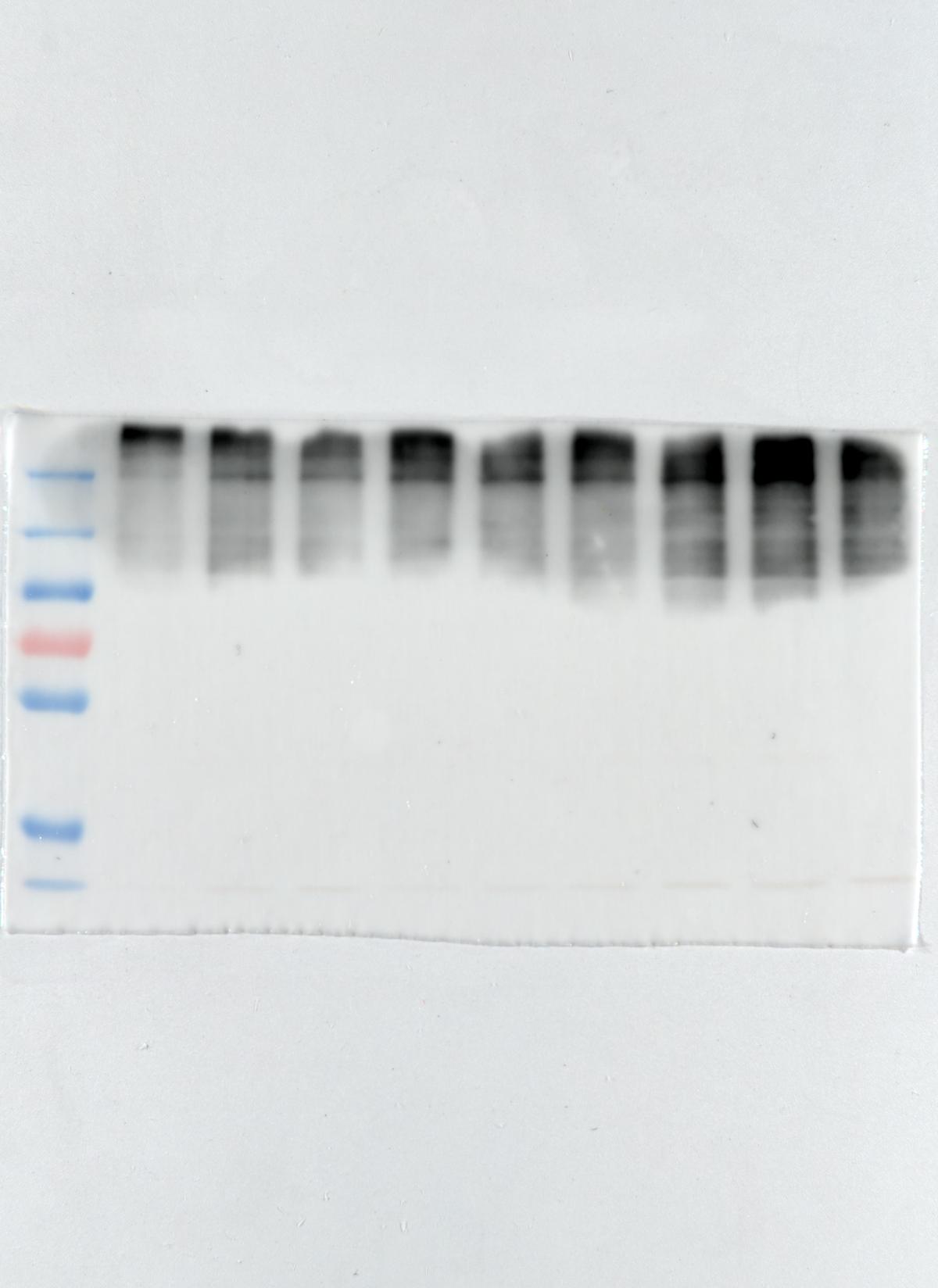

Supplement: Supplementary file 1 [file nutrients-17-02431-s001.zip › colon-WB/7.4 zo1 20240709_102519_Ch/7.4 zo1 20240709_102519_Ch_Chemi+Marker.jpg]

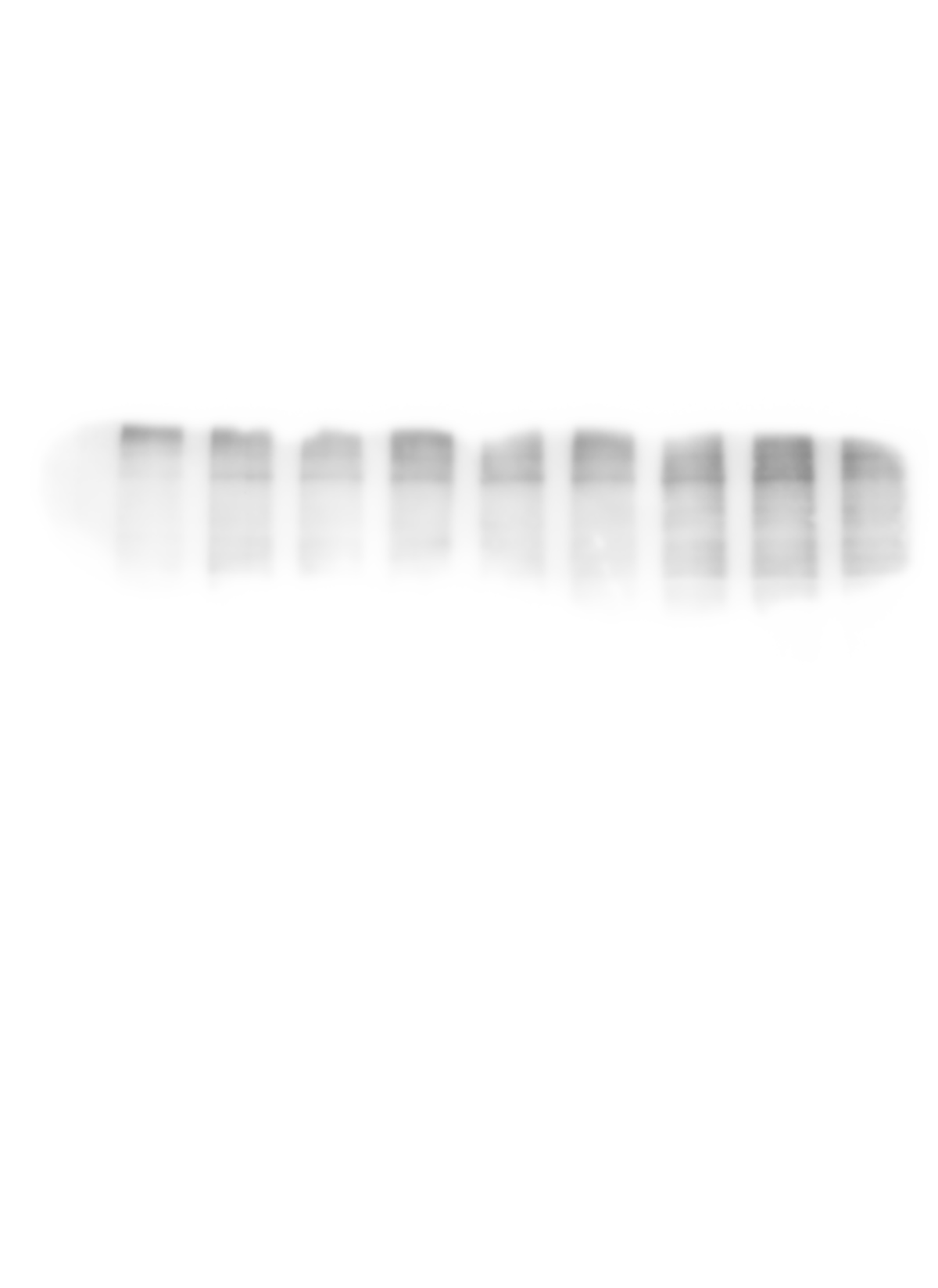

Supplement: Supplementary file 1 [file nutrients-17-02431-s001.zip › colon-WB/7.4 zo1 20240709_102519_Ch/7.4 zo1 20240709_102519_Ch_Chemi.tif]

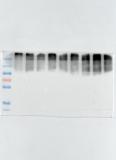

Supplement: Supplementary file 1 [file nutrients-17-02431-s001.zip › colon-WB/7.4 zo1 20240709_102519_Ch/7.4 zo1 20240709_102519_Ch_Thumb.jpg]

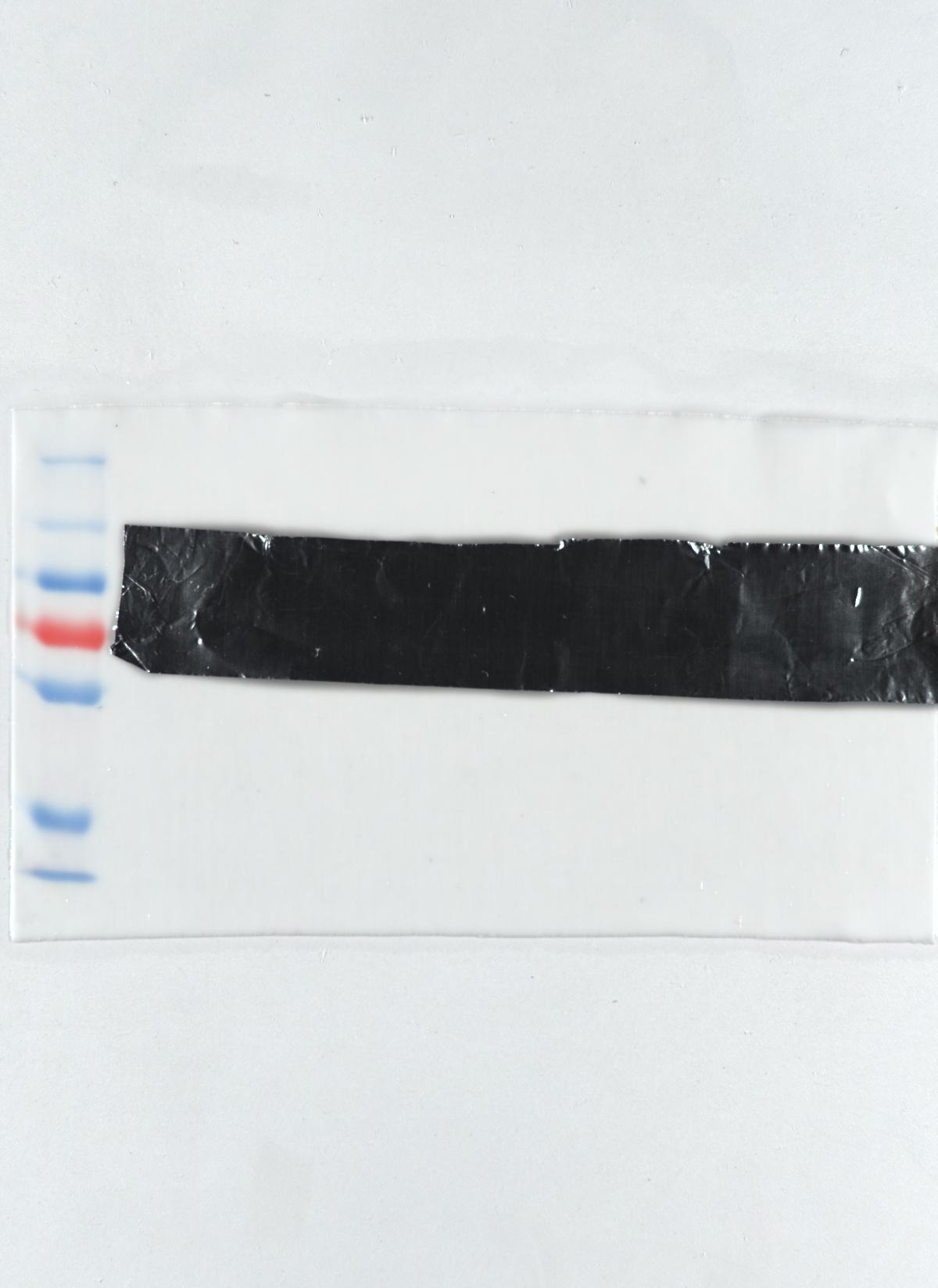

Supplement: Supplementary file 1 [file nutrients-17-02431-s001.zip › colon-WB/7.4 zo1× 20240704_004546_Ch/7.4 zo1× 20240704_004546_Ch-Marker.jpg]

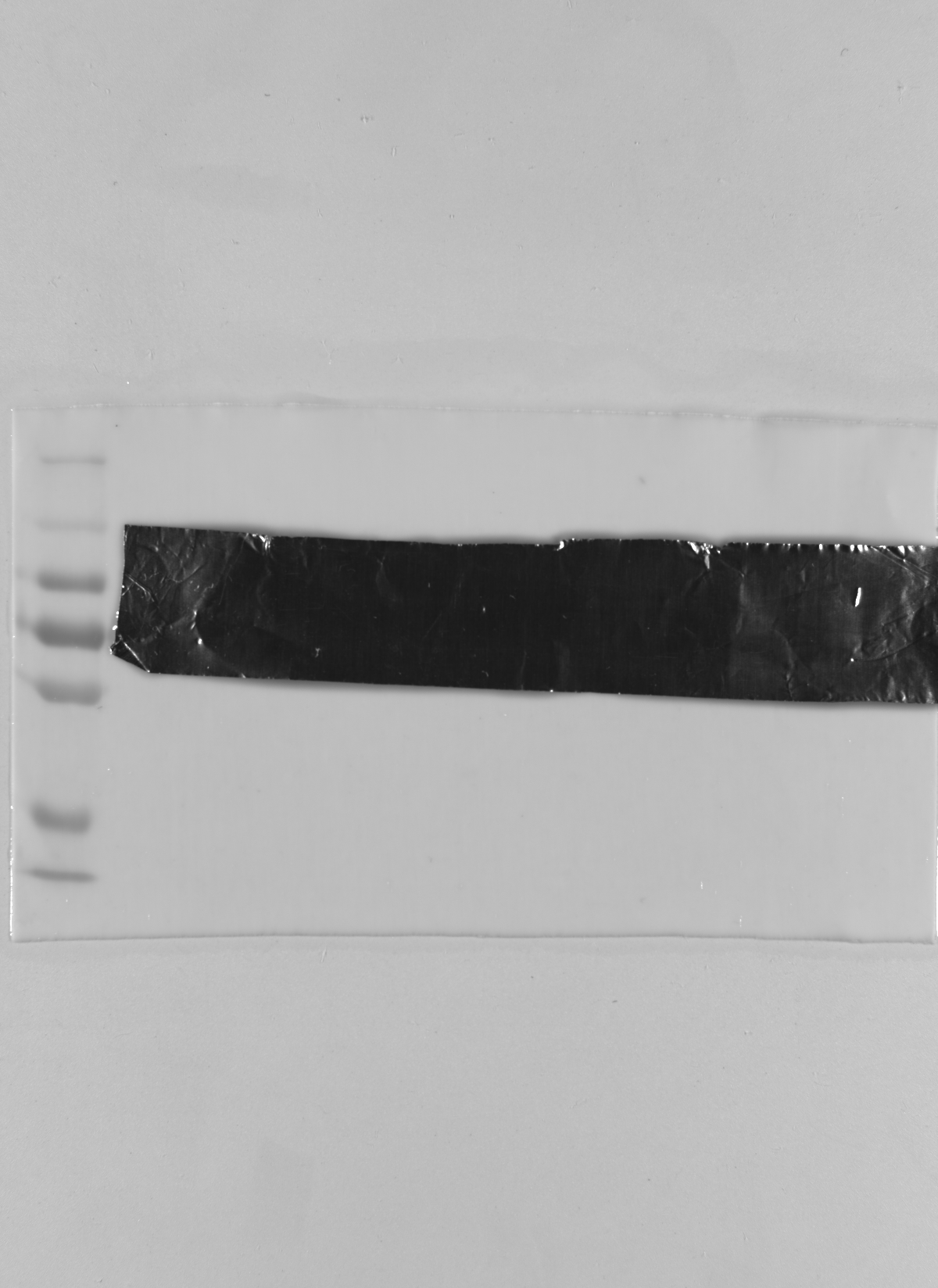

Supplement: Supplementary file 1 [file nutrients-17-02431-s001.zip › colon-WB/7.4 zo1× 20240704_004546_Ch/7.4 zo1× 20240704_004546_Ch-Marker.tif]

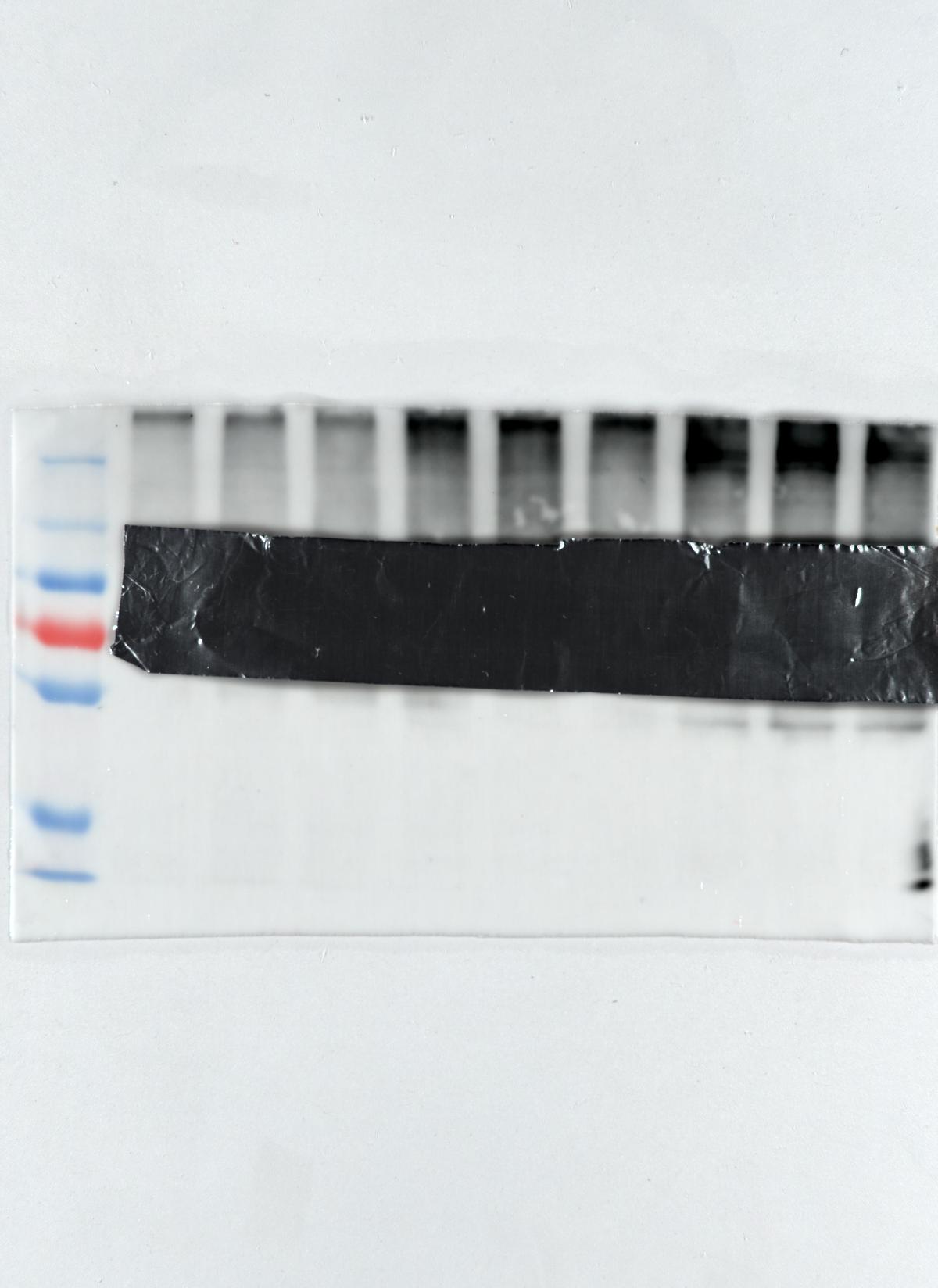

Supplement: Supplementary file 1 [file nutrients-17-02431-s001.zip › colon-WB/7.4 zo1× 20240704_004546_Ch/7.4 zo1× 20240704_004546_Ch_Chemi+Marker.jpg]

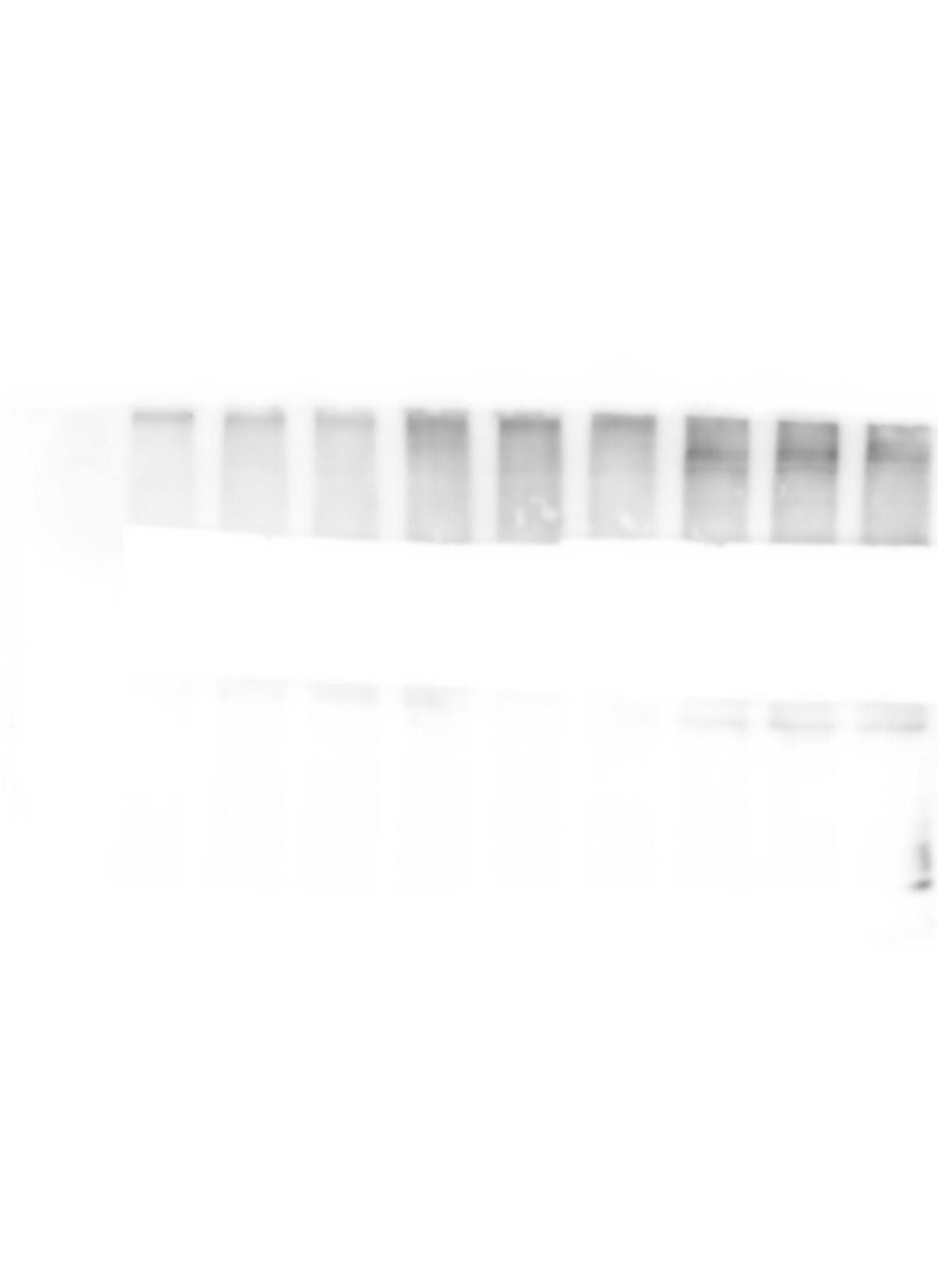

Supplement: Supplementary file 1 [file nutrients-17-02431-s001.zip › colon-WB/7.4 zo1× 20240704_004546_Ch/7.4 zo1× 20240704_004546_Ch_Chemi.tif]

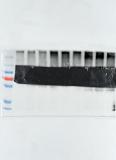

Supplement: Supplementary file 1 [file nutrients-17-02431-s001.zip › colon-WB/7.4 zo1× 20240704_004546_Ch/7.4 zo1× 20240704_004546_Ch_Thumb.jpg]
